# Thermal Safety Margins and Peak Leaf Temperatures Predict Vulnerability of Diverse Plant Species to an Experimental Heatwave

**DOI:** 10.1111/pce.70041

**Published:** 2025-06-29

**Authors:** Diana Cox, Renée M. Marchin, David S. Ellsworth, Agnieszka Wujeska‐Klause, Alessandro Ossola, Kristine Y. Crous, Michelle R. Leishman, Paul D. Rymer, Mark G. Tjoelker

**Affiliations:** ^1^ Hawkesbury Institute for the Environment Western Sydney University Penrith New South Wales Australia; ^2^ Urban Transformations Research Centre Western Sydney University Parramatta New South Wales Australia; ^3^ Department of Biological Sciences Macquarie University North Ryde New South Wales Australia; ^4^ Department of Plant Sciences University of California Davis Davis California USA

**Keywords:** heat tolerance, leaf critical temperature, maximum leaf temperature, thermal acclimation, thermal plasticity

## Abstract

Extreme heat can push plants beyond their thermal safety margin (*TSM*) if maximum leaf temperature (*T*_*leaf_max*_) exceeds leaf critical temperature (*T*_*crit*_). The *TSM* is potentially useful for assessing heat vulnerability across species but needs further validation, so we exposed 50 tree/shrub species in controlled glasshouses to a 6‐day heatwave (peak air temperature = 41°C). Many species increased their mean *T*_*crit*_ during the heatwave (42%), with Δ*T*_*crit*_ ranging from +1°C to 4°C, but other species did not acclimate or were impaired by heat stress (58%). Species *T*_*leaf_max*_ explained ~55% of the variation in species *T*_*crit*_ and was a key correlate of the plasticity of *T*_*crit*_ among species. Species with high Δ*T*_*crit*_ also had higher Δ*T*_*leaf_max*_, with leaves being 7°‒12°C hotter during the heatwave than under baseline conditions. Both *T*_*leaf_max*_ and *TSMs* were correlated with heatwave damage across diverse species from contrasting climate zones. Species differences in *TSMs* were stable across measurement temperatures, correctly identified the most vulnerable species, and were strongly associated with *T*_*leaf_max*_. Our results suggest that (1) *T*_*leaf_max*_ alone is more informative than *T*_*crit*_ for ranking species heat tolerance, and (2) species vulnerability to heatwaves is most reliably assessed by using *TSMs* that integrate *T*_*leaf_max*_ with *T*_*crit*_ across species.

## Summary statement

Thermal safety margin was a reliable predictor of plant species vulnerability to an experimental heatwave. Leaf temperature, and not air temperature, was a key correlate of heat tolerance differences among species and was also related to the degree of short‐term acclimation of heat tolerance.

## 1 Introduction

Heatwaves are becoming more frequent and intense (IPCC 2022; Perkins‐Kirkpatrick and Lewis 2020), which threatens plant survival by inducing heat stress and leaf damage, especially when heatwaves occur in combination with other stress factors (Breshears et al. 2021; Hammond et al. 2022; Marchin et al. 2022). Some plant species exhibit constitutive high thermal tolerance of heat stress, whereas others exhibit induced responses and short‐term acclimation after exposure to high temperatures (Guilioni 1997; Vollenweider and Günthardt‐Goerg 2005; Wahid et al. 2007). Heat adaptation often involves leaf traits that confer tolerance or avoidance of high heat load through modifications of leaf energy balance (He et al. 2018) that might also show phenotypic plasticity in response to heat events. The role of intrinsic differences in acclimation of thermal tolerance to heat stress exposure remains poorly understood across plant species.

Heatwaves can cause heat stress in terrestrial plants when temperatures exceed the optimal range for plant function, resulting in a cascade of physiological and metabolic responses (Teskey et al. 2015; Wahid et al. 2007). In leaves, the photosynthetic machinery is adversely affected by excessive heat (Takahashi and Murata 2008), leading to a reduction of the potential quantum efficiency of photosystem II (PS II; Krause and Weis 1991; Maxwell and Johnson 2000). Heat stress can be manifested in altered membrane stability, degradation of proteins, inactivation of enzymes or changing water relations (Wahid et al. 2007). Stress effects are often reversible until a critical leaf temperature threshold is reached (Cunningham and Read 2006), after which permanent damage occurs (Bita and Gerats 2013; Knight and Ackerly 2003; Sage and Kubien 2007). The critical leaf temperature threshold above which damage to PS II begins to accumulate is termed *T*_*crit*_ and can be measured via the temperature‐dependent rise in chlorophyll *a* fluorescence (Bilger et al. 1984; Knight and Ackerly 2002). The *T*_*crit*_ is directly related to the high‐temperature stability of chloroplast membranes and membrane‐bound proteins (Allakhverdiev et al. 1996; Bukhov et al. 1999; Havaux 1993) and is a common measure of the heat tolerance of plants (Bilger et al. 1984; Geange et al. 2021; Knight and Ackerly 2003; Krause et al. 2013; O'Sullivan et al. 2013, 2017; Taub et al. 2000).

There is high plasticity in *T*_*crit*_ both within and across species due to predictable changes in temperature over time, such as diurnal and seasonal temperature changes, but also due to stochastic events like heatwaves that can enhance plant heat tolerance (Havaux and Tardy 1996; Krause et al. 2010, 2013; O'Sullivan et al. 2013; Seemann et al. 1984; Zhu et al. 2018). Studies have shown that seasonal changes in *T*_*crit*_ range from 4°C to 9°C, corresponding to a 4°C to 12°C change in seasonal air temperature (Kullberg and Feeley 2024; Seemann et al. 1986; Zhu et al. 2018). Plasticity of *T*_*crit*_ after exposure to high temperatures is complex, varying with short‐term heat pulses (15 min to 2 h; Curtis et al. 2014; Havaux and Tardy 1996; Krause et al. 2010), hot days followed by cold nights (Harris et al. 2024), longer multi‐day heatwaves (Drake et al. 2018) and successive heatwaves in summer (Ahrens et al. 2021). These *T*_*crit*_ increases are thought to occur due to changes in lipid membrane composition (Hochachka and Somero 2002; Zhu et al. 2018), increases of cellular thermo‐protectant osmolytes (Hüve et al. 2006; Niinemets 2018), and/or heat shock proteins (Vierling 1991). Taxa originating in temperate or arid climates with larger seasonal changes in temperature might be expected to exhibit greater plasticity in *T*_*crit*_ than tropical origin taxa. Whether plasticity in *T*_*crit*_ is phylogenetically dependent (i.e., the tendency of more closely related species to exhibit similar plasticity in *T*_*crit*_ values), or differs among climate zones, requires further testing.

Thermal safety margins (*TSMs*) quantify how close peak maximum leaf temperature (*T*_*leaf_max*_) is to reaching *T*_*crit*_ under a given set of environmental conditions (O'Sullivan et al. 2017; Perez and Feeley 2020) and have been used to infer species vulnerability to heatwaves. Species with higher *TSMs* are typically expected to be less vulnerable to damage during heatwaves, relative to species with lower *TSMs* (Marchin et al. 2022; O'Sullivan et al. 2017), although this has rarely been explicitly tested. Photosynthetic heat tolerance does not always correlate positively with *TSM*, as Perez and Feeley (2020) found a negative relationship between heat tolerance and *TSM* for 19 tropical species in a common garden. This suggests species with higher heat tolerance may actually be more susceptible to thermal damage or simply operating closer to their upper thermal thresholds (Slot et al. 2021). Tropical species have evolved under stable climatic conditions, tend to have narrower *TSMs* (Kitudom et al. 2022), and may be approaching their critical temperature thresholds (Doughty et al. 2023; Tiwari et al. 2021). Another complicating factor is that various definitions for *TSM* have been used across studies, including the use of air, canopy or leaf temperature. Using *TSM* calculated based on air temperature, since leaf or canopy temperature is difficult to measure in tall trees or across large areas, O'Sullivan et al. (2017) and Sastry and Barua (2017) inferred that climate warming is increasing species vulnerability to heat stress. Using leaf rather than canopy or air, temperature for *TSM* is more physiologically relevant when determining vulnerability to heat stress in plants (Cook et al. 2021; Manzi et al. 2024). Further study of *TSMs* across plant species is needed to develop the *TSM* concept and provide a better understanding of the thermal sensitivity of different tree species.

In this study, we grew 50 diverse tree and shrub species from contrasting climate zones under controlled conditions in glasshouses to determine the impact of a 6‐day summer heatwave (peak temperature of 41°C) on high‐temperature acclimation of *T*_*crit*_ and *TSM*. A major aim of our study was to rank the heat tolerance of common horticultural species, plus novel species that may be suited for urban plantings, to help improve the performance and survival of urban trees under a changing climate. Our study addressed the following questions:

1. Do species differ in acclimation of *T*_*crit*_ to heatwave exposure, and if so, are species differences related to phylogeny or climate zone?
2. To what extent are species differences in *T*_*leaf_max*_, and leaf versus air temperature (*T*_*diff*_), related to their heat tolerance (*T*_*crit*_) and ability to acclimate to heatwaves (Δ*T*_*crit*_)?
3. Are *TSMs* that use *T*_*leaf_max*_ and *T*_*crit*_ to integrate whole‐plant responses to high temperature a reliable measure for ranking species vulnerability to heatwaves, and if so, are species differences more related to *T*_*leaf_max*_ or *T*_*crit*_?

We hypothesised that (1) *T*_*crit*_ will exhibit short‐term thermal acclimation to heatwave exposure, (2) *T*_*crit*_ differences among species are related to their *T*_*leaf_max*_ and (3) *TSMs* based on peak leaf temperature are a reliable parameter for ranking species vulnerability to heatwaves.

## 2 Materials and Methods

### 2.1 Study Species and Glasshouse Experiments

We selected 50 tree and shrub species across a large phylogenetic spectrum, including many horticultural species, both native and exotic to Australia, with varied leaf phenology (evergreen to deciduous, Table S1). The species' native climate classification encompassed four different Köppen–Geiger climate zones: arid, tropical, subtropical and temperate. Information regarding each species' accepted native range in *Kew Backbone Distributions* (KBD 2020) was retrieved from *Plants of the World Online* (POWO 2019). Occurrence records of each species were assessed via *Global Biodiversity Information Facility* (GBIF 2019). Each species was classified into a single climate zone based upon the majority distribution of occurrence records, spanning habitats from arid woodlands to rainforests (Table S1). Occurrence records were excluded when coordinates were missing or erroneous (e.g., located in water bodies) or if records were older than 1950 (deemed unreliable; Burley et al. 2019). Occurrences outside of the native range were manually removed with an open‐source geographic information system (QGIS 2019).

Planting stock was purchased from commercial nurseries as tube stock (68 mm wide and 125 mm deep or 90 mm wide and 150 mm deep). The plants (*n* = 5 plants per species) were potted up individually into 6 L pots (diameter 183 mm, depth 240 mm). The substrate consisted of a potting mix of 30% coir/sand and 70% screened composted pine bark by volume (Australian Growing Solutions, Tyabb, VIC, Australia), a slow‐release fertiliser (38 g per pot; Scotts Australia Osmocote Slow Release, Bella Vista, NSW, Australia), and a systemic insecticide and fertiliser tablet (1.25 g per pot, Yates Confidor, Padstow, NSW, Australia).

Plants were grown in two separate controlled‐environment glasshouse locations in Sydney, New South Wales, Australia, due to space limitations. The experiments spanned the austral mid‐spring to mid‐autumn period (October–April). Twenty‐four species were grown in a glasshouse at the Hawkesbury Institute for the Environment (HIE; Western Sydney University, Richmond, NSW) from 1 October 2018 to 12 March 2019 in two bays under natural sunlight (91% transmittance, UV‐inclusive, Plexiglas Alltop SDP 16/980 – 64, Evonik Industries AG, Germany). Twenty‐six species were grown in a glasshouse at the Plant Growth Facility (MQ; Macquarie University, North Ryde, NSW) from 10 December 2018 to 18 April 2019 in three bays under natural sunlight (82% transmittance, UV excluded < 385 nm wavelengths, Lexan Thermoclear sheet – LT2UV62RS13, SABIC Innovative Plastics, China). Air temperature and relative humidity were similar across the two glasshouse locations, but mean and maximum photosynthetically active radiation were higher in the HIE than the MQ glasshouse (Table S2) due to the differences in glasshouse materials described above. As a result, *T*_*leaf_max*_ (*Χ* ^2^ = 14.06, *p* < 0.001) and *T*_*diff*_ (*Χ* ^2^ = 16.67, *p* < 0.001) were also higher in the HIE than MQ glasshouse, but there was no difference in *T*_*crit*_ (*p* = 0.25) between the two locations (Figure S1).

To minimise the potential effects of spatial heterogeneity in growing conditions within each location, the plants were regularly rotated within and between bays. In both glasshouse locations, the air temperature of growing conditions was set to a daily mean of 27°C, with a six‐step temperature profile from 21°C to 34°C (Table S3) to mimic natural diurnal patterns (Poorter et al. 2016). The glasshouse daytime relative humidity was set to 60% to 80%. Each pot was connected to automatic drip irrigation and kept well‐watered at a soil volumetric water content of > 30%, which was monitored regularly using time‐domain reflectometry.

During the baseline growth phase, all plants (*n* = 5 pots per species) were grown for at least 6 weeks to enable growth and production of new leaves under glasshouse conditions. After this initial growth period, separate groups of 25 or 30 plants (i.e., batches of five or six species) were exposed to a 6‐day heatwave. Average peak air temperature of the heatwave was 41°C from 12:00 to 14:00, +7°C above the baseline temperature of 34°C, implemented in a seven‐step diurnal temperature profile ranging from 30°C to 41°C (Table S3). Baseline physiological measurements (see below) were conducted in the week before the experimental heatwave. Measurements during the heatwave were collected on Days 5 or 6, or in the case of *T*_*leaf_max*_, on a single sunny day. All measurements were conducted on recently developed and fully expanded leaves.

### 2.2 Leaf Critical Temperature

Leaf critical temperature (*T*_*crit*_) was determined in vivo on dark‐adapted leaves. The measurement system (Marchin et al. 2022) was based on the method of Schreiber and Berry (1977) and consisted of a Mini‐PAM fluorometer (Mini‐PAM model II/B; Walz GmbH, Effeltrich, Germany), a temperature controller (Arduino PID temperature controller, Monza, IT), and a mini‐computer (Raspberry Pi 3B with touch screen, Core Electronics, Kotara, NSW, Australia). The attached leaves were dark‐adapted for a minimum of 10 min at 35°C. After stability was reached, minimum fluorescence (*F*_o_) was recorded every second as temperature was gradually increased by 0.5°C every 30 s until maximum fluorescence (*F*_m_) was reached. The *T*_*crit*_ was calculated as the interception point of the linear interpolation of slow‐ and fast‐rise phases in fluorescence with temperature (Bilger et al. 1984; Krause et al. 2010; Valladares and Pearcy 1997). *T*_*crit*_ was measured on one sun‐exposed leaf per plant (*n* = 3–5 plants per species), except for *Eucryphia lucida* and *Pittosporum bicolor*, which showed high levels of visible injury and chlorosis after the heatwave and hence could not be measured. The Δ*T*_*crit*_ was calculated for 48 of 50 species by subtracting baseline *T*_*crit*_ from heatwave *T*_*crit*_ for each individual plant.

### 2.3 Leaf Temperature and TSMs

Peak maximum leaf temperature (*T*_*leaf_max*_) was measured on three leaves per plant with a handheld infrared thermometer (Agri_Therm III Model 6110 L, Everest Interscience Inc., Chino Hills, OR, USA). Baseline and heatwave data were obtained from fully developed leaves, and where possible, midribs of broad leaves were avoided. All measured leaves were exposed to full‐sun conditions in the glasshouse for at least 10 min before measurement. Measurement occurred on sunny days between 12:00 and 14:00 when air temperature peaked (34°C baseline, 41°C heatwave). Three *T*_*leaf_max*_ values per plant were averaged for five plants per species (*n* = 15 leaves per species). The Δ*T*_*leaf_max*_ was calculated by subtracting the baseline *T*_*leaf_max*_ from heatwave *T*_*leaf_max*_, and the difference (*T*_*diff*_) between air temperature and *T*_*leaf_max*_ was also calculated. Due to the requirement for full‐sun conditions, the occurrence of cloudy days resulted in missing values, and we obtained complete baseline and heatwave *T*_*leaf_max*_ for 34 of 50 species. TSMs (°C) were calculated separately for baseline and heatwave conditions for each plant as:

$$\mathrm{TSM}=T_{\mathrm{crit}}-T_{\mathrm{leaf}\_\max}.$$

### 2.4 Plant Damage Indicators

Damage indicators of photosystem function in response to heat exposure are used to infer heat tolerance (Curtis et al. 2014; Geange et al. 2021). We measured two damage indicators: the maximum quantum yield of PS II (*F*_*v*_ */F*_*m*_) and foliar leaf injury. To assess *F*_*v*_ */F*_*m*_, variable (*F*_*v*_) and maximum chlorophyll *a* fluorescence (*F*_*m*_) were measured with the mini‐PAM II/B (Walz GmbH, Effeltrich, Germany) on one dark‐adapted leaf per plant using dark adaptation leaf clips (DLC‐8; Walz GmbH, Effeltrich, Germany). Leaves were selected and flagged to permit a repeat comparison under baseline and heatwave conditions. Before *F*_*v*_ */F*_*m*_ measurement, the leaves were dark‐adapted for a minimum of 30 min. After measuring *F*_o_, a saturation pulse was applied to measure maximum fluorescence (*F*_m_).

Visible injury, including chlorotic lesions, leaf scorch and browning, results from heat damage and has been previously used to quantify heat‐induced leaf necrosis (Bilger et al. 1984). Visible injury arising from heatwave exposure was defined as the percentage of total leaf area exhibiting browning or scorching on each plant. Measurements were conducted independently by two observers and then averaged for each plant. Visible injury was assessed the week before the heatwave (baseline), at the end of the heatwave (heatwave), and 2 weeks after the heatwave (recovery). Independent injury assessments of two observers were correlated for baseline (*r* = 0.81, *p* < 0.001), end of heatwave (*r* = 0.86, *p* < 0.001), and after 2 weeks (*r* = 0.90, *p* < 0.001). Maximum injury (either heatwave or recovery value) was selected, from which baseline levels were subtracted to calculate maximum heatwave damage.

### 2.5 Phylogenetic Analysis

Taxonomic information was obtained with the ‘classification’ function from the R package *taxize* (Chamberla and Szocs 2013) from the National Centre for Biotechnology Information (NCBI) database. An ultra‐metric tree with 47 of 50 species from 25 families, with a smoothing parameter of 1 and a correlated substitution model, was created (*chronos* from *ape* package; Kim and Sanderson 2008). The hybrid cultivars, *Photinia × robusta* and *Platanus × acerifolia*, were excluded from analysis. Another species (*Pomaderris apetala*) was excluded as taxonomic information was not available in the NCBI database. The phylogenetic signal of Δ*T*_*crit*_ was assessed using Blomberg's *K* (*phylosignal* from *picante* package; Blomberg and Garland 2002).

### 2.6 Statistical Analysis

All statistical analyses were performed with R (version 3.5.1; R Core Team 2021). The effect of the heatwave on *T*_crit_ and *TSM* was determined using full‐factorial, mixed‐model analyses of variance (ANOVAs; *ANOVA* from car package; Fox and Weisberg 2019) with species and treatment as the main effects; species identity was included as a random effect and treatment as a fixed effect. Separate models were used to test for species × treatment interactions, and if significant (*p* ≤ 0.05), differences among species were analysed using Student's *t*‐tests. Species differences in the acclimation of *T*_*crit*_ (Δ*T*_*crit*_) between baseline and heatwave conditions were assessed using a linear model (*lm* from *stats* package) in which species was a fixed factor. To test for a possible effect of climate zone on Δ*T*_*crit*_, climate zone was considered a fixed factor and analysed with a linear mixed effects model (*lmer* from *lme4* package), where species was a random factor. To test for potential glasshouse differences in *T*_*crit*_, *T*_*leaf_max*_ and *T*_*diff*_, an ANOVA was used in which glasshouse location and heatwave treatment were used as fixed factors and species as a random factor.

Linear models were used to test the relationships between *T*_*crit*_ and *T*_*leaf_max*_ or *T*_*diff*_ with heatwave treatment as a fixed factor. Analysis of covariance (ANCOVA) was used to test for differences in slope and intercept between baseline and heatwave treatment groups (Chambers and Hastie 1992). A linear model was also used to examine the relationship between changes in *T*_*crit*_ (Δ*T*_*crit*_) and the difference in *T*_*leaf_max*_ (Δ*T*_*leaf_max*_) between baseline and heatwave treatments for all species pooled across both glasshouse locations. Three species (*Atractocarpus benthamianus*, *Syzygium anisatum*, *Syzygium wilsonii*) exhibited comparatively high levels of visible leaf injury, low *F*_*v*_ */F*_*m*_, and large reductions in *T*_*crit*_ under heatwave conditions, indicating excessive heat damage; these species were excluded from analysed relationships between *T*_*crit*_, *T*_*leaf_max*_ or *T*_*diff*_, and *TSM*.

To test if damage (*F*_*v*_ */F*_*m*_ or visible injury) across species was related to their *TSM* or *T*_*leaf_max*_, generalised additive models (*gam* from *mgcv* package; Wood et al. 2016) with a Gaussian family and cubic spline smoothing term were used. The correlation between baseline *TSM* and heatwave *TSM* was assessed with a Spearman rank correlation, while the correlation between damage indicators *F*_*v*_ */F*_*m*_ and leaf visible injury was tested with Pearson's correlation test (*cor. test* from *stats* package). Linear models were used to examine relationships between species' TSM and the two *TSM* components (i.e., *T*_*crit*_, *T*_*leaf_max*_).

Data were checked for normality using Shapiro–Wilk's test (*shapiro. test* from *stats* package; Royston 1995) and by inspecting the distribution density and Q–Q plots. Homogeneity of variance was confirmed using Levene's test (*leveneTest* from car package; Fox and Weisberg 2019) and by inspection of the standardised residual plots. The *T*_*crit*_ and calculated *TSM* were normally distributed, while *F*_*v*_ */F*_*m*_ was transformed with Tukey's ladder of power transformation (*transformTukey* from *rcompanion* package; Mangiafico 2016) to achieve normality. No transformation for *T*_*leaf_max*_ was found to meet requirements for a normal distribution, so non‐parametric tests were used. All statistical tests with *p*‐values < 0.05 were considered statistically significant. Errors are expressed as standard errors of the mean (SE).

## 3 Results

### 3.1 Species Acclimation of *T*_*crit*_ After Heatwave Exposure

**Figure 1:**
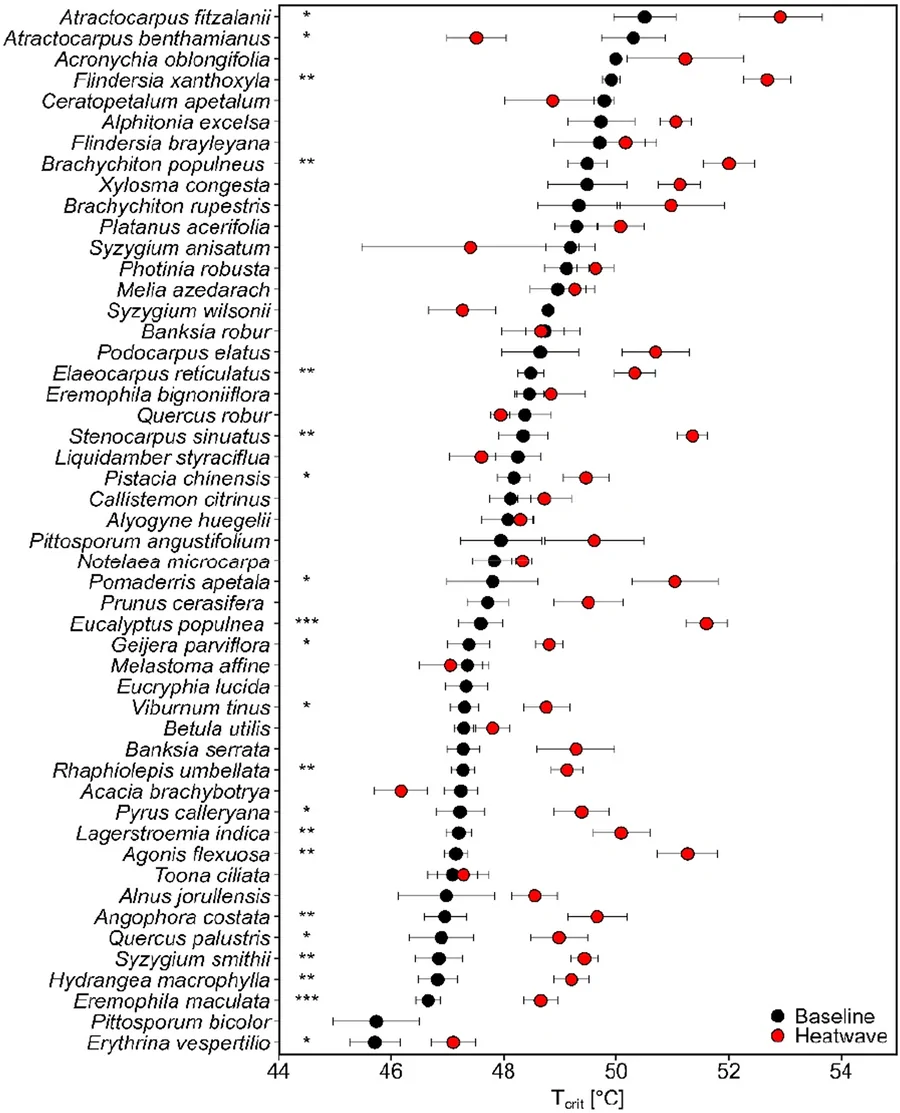
Heat tolerance (*T*_*crit*_) of 50 tree or shrub species before (baseline, black) and during a 6‐day 41°C heatwave (red). The species are ordered according to their mean baseline *T*_*crit*_ value (*n* = 3–5 plants per species). Two species (*Eucryphia lucida*, *Pittosporum bicolor*) are missing heatwave values due to leaf loss during the heatwave. Error bars represent SE. Asterisks denote acclimation of *T*_*crit*,_ during the heatwave according to Student's *t*‐tests: ***p < 0.001; **p < 0.01; *p < 0.05.

Most species (39 out of 48 species, 81%) increased mean *T*_*crit*_ following 6 days of +7°C heatwave treatment, relative to baseline values, although the increase was only significant for about half of those species (20 species; Figure 1, Table S4). The magnitude of *T*_*crit*_ acclimation ranged from 1.3°C to 4.0°C (Figure 1). There were large differences in Δ*T*_*crit*_ among species (*F*_47,143_ = 3.5, *p* < 0.001), ranging from −2.4 ± 1.1°C to 4.0 ± 0.6°C with a mean Δ*T*_*crit*_ of 1.2 ± 0.1°C across all 48 species (Figure S2). Nine out of 48 species (19%) had mean Δ*T*_*crit*_ < 0°C, although the decrease was only significant for one species (*A. benthamianus*), plus two species could not be measured due to heat damage and leaf loss during the heatwave (*E. lucida*, *P. bicolor*; Figure 1, Table S4). A total of 27 species had no significant change in mean *T*_*crit*_ after the heatwave (Figure 1, Table S4). Species groups based on native climate zones did not differ in Δ*T*_*crit*_ (*F*_3,191_ = 0.57, *p* = 0.64). We did not observe a phylogenetic signal related to species differences in Δ*T*_*crit*_ (Blomberg's *K* = 0.05, *p* = 0.68).

### 3.2 Testing If Temperature Influences *T*_*crit*_ and Δ*T*_*crit*_ Variation Among Species

**Figure 2:**
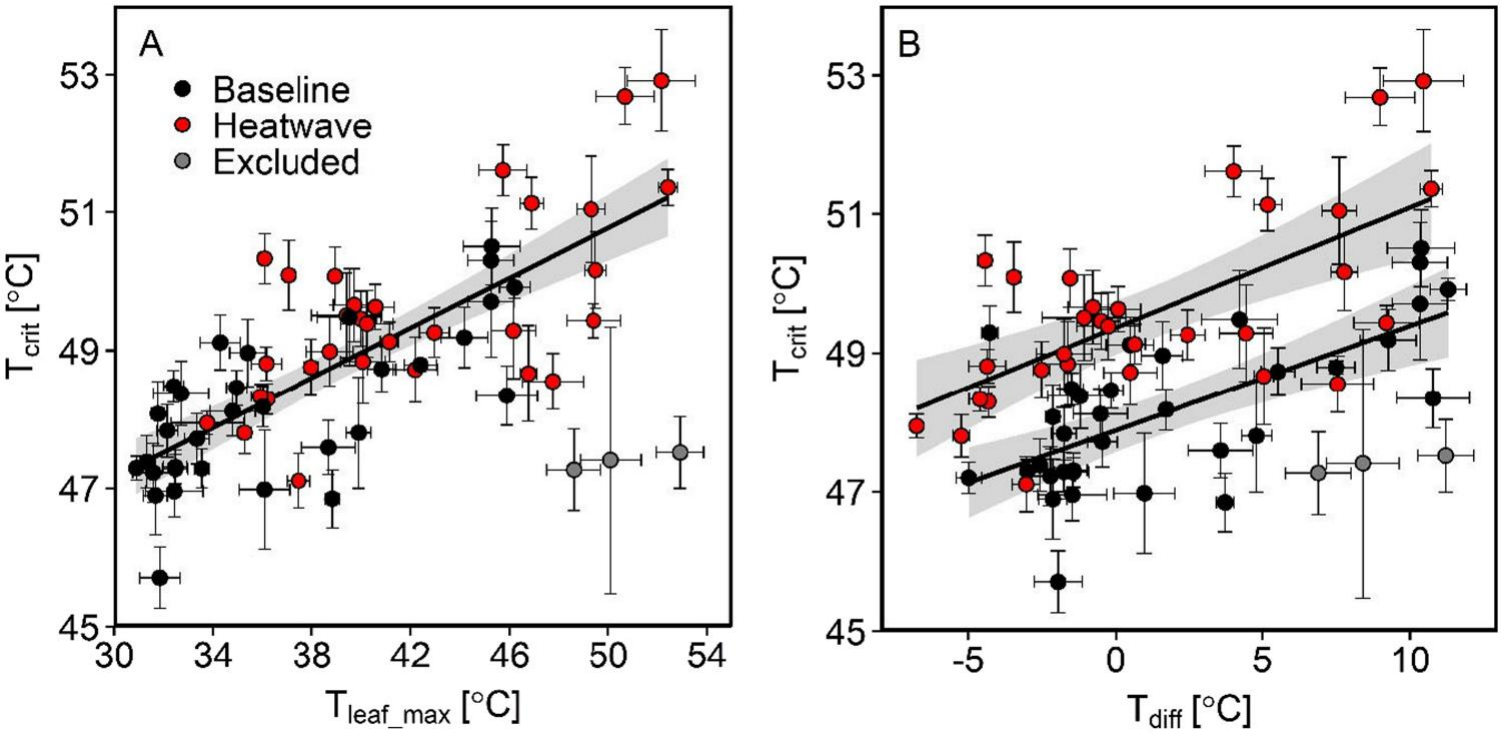
(A) Heat tolerance (*T*_*crit*_) was linearly related to the maximum leaf temperature (*T*_*leaf_max*_) of 34 tree or shrub species measured under baseline and +7°C heatwave conditions; *T*_*crit*_ = 42.66 + 0.16 × *T*_*leaf_max*_ (*r* ^2^ = 0.56, *p* < 0.001, *n* = 65). (B) *T*_*crit*_ was linearly related to the difference between *T*_*leaf_max*_ and air temperature (*T*_*diff*_) during baseline: *T*_*crit*_ = 47.88 + 0.15 × *T*_*diff*_ (*r* ^2^ = 0.44, *p* < 0.001, *n* = 34) and heatwave conditions: *T*_*crit*_ = 49.21 + 0.09 × *T*_*diff*_ (*r* ^2^ = 0.44, *p* < 0.001, *n* = 31), where positive values indicate leaves are hotter than air and negative values indicate leaves are cooler than air. All values are species means (±SE). The three grey points were excluded from analysis, owing to heat damage to *Atractocarpus benthamianus*, *Syzygium anisatum* and *Syzygium wilsonii*. [Color figure can be viewed at wileyonlinelibrary.com]

Given evidence of short‐term acclimation of *T*_*crit*_ to heatwave temperatures, we examined species differences in maximum leaf temperature (*T*_*leaf_max*_) and leaf versus air temperature differences (*T*_*diff*_) as potential correlates of *T*_*crit*_ under baseline and heatwave conditions (Figure 2). Species mean *T*_*crit*_ increased with increasing mean *T*_*leaf_max*_, exhibiting a common positive linear relationship across all species under baseline and heatwave treatments combined (*F*_2,62_ = 19.18, *r* ^2^ = 0.56, *p* < 0.001, *n* = 65; Figure 2A). There were no differences in the slope (*p* = 0.52) or intercept between the linear models of *T*_*crit*_ and *T*_*leaf_max*_ for baseline versus heatwave treatments (*p* = 0.09).

**Figure 3:**
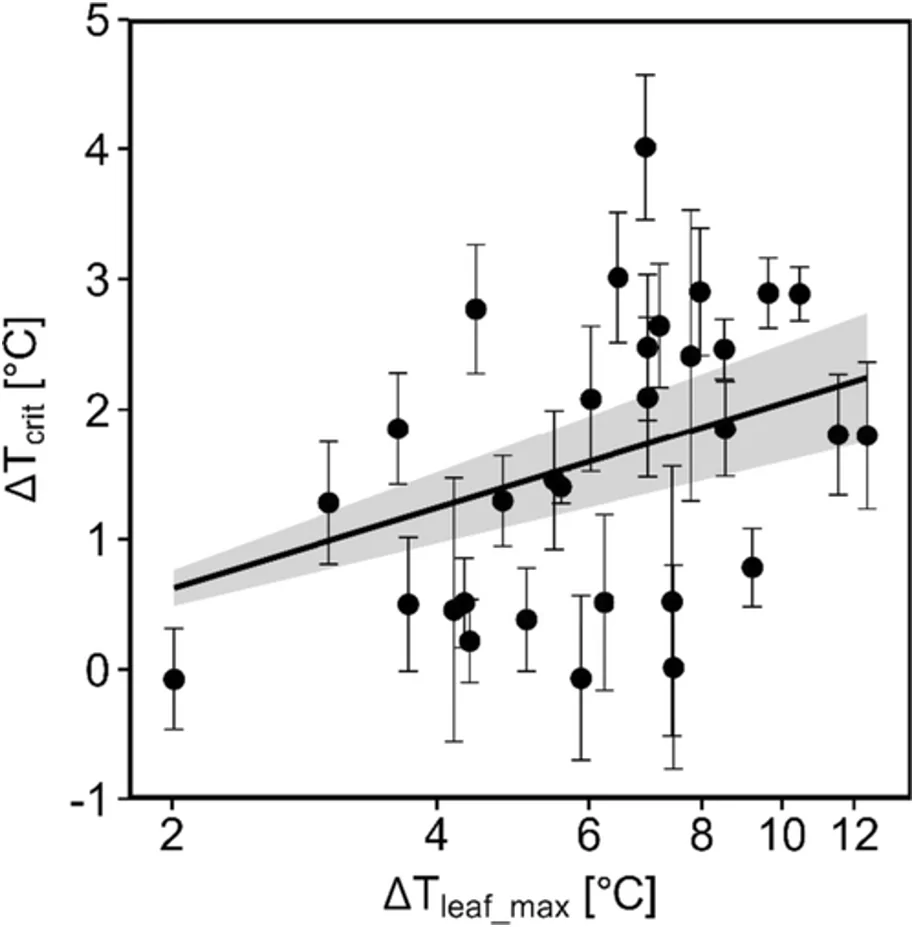
Relationship between short‐term acclimation of heat tolerance (Δ*T*_*crit*_) to a +7°C heatwave and change in maximum leaf temperature (Δ*T*_*leaf_max*_), relative to baseline conditions, for 31 tree or shrub species. The fitted log‐linear relationship (*r* ^2^ = 0.21, *p* = 0.01): Δ*T*_*crit*_ = 1.24 × ln(Δ*T*_*leaf_max*_). Points represent species means (±SE). Note that the Δ*T*_*leaf_max*_ *x*‐axis is expressed on a natural log scale.

The overall range in mean *T*_*diff*_ values among species was comparable between baseline and heatwave conditions, indicating that heatwave exposure did not systematically increase mean *T*_*diff*_ (*p* = 0.44). Mean *T*_*crit*_ increased linearly with increasing mean *T*_*diff*_ across species in both baseline (*r* ^2^ = 0.44, *p* < 0.001, *n* = 34) and heatwave conditions (*r* ^2^ = 0.44, *p* < 0.001, *n* = 31; Figure 2B). Species mean *T*_*crit*_ values were higher in heatwave than baseline conditions for the measured range of *T*_*diff*_, indicating that *T*_*diff*_ was consistently related to observed *T*_*crit*_ differences among species between baseline and heatwave conditions (*F*_2,62_ = 19.24, *p* < 0.001; Figure 2B). Further inspection of mean Δ*T*_*crit*_ across species in relation to changes in mean *T*_*leaf_max*_ in heatwave versus baseline conditions (Δ*T*_*leaf_max*_) revealed a positive log‐linear relationship (*F*_1,29_ = 7.57, *r* ^2^ = 0.21, *p* = 0.01, *n* = 31; Figure 3).

### 3.3 TSMs and Species Vulnerability to Heatwaves

**Figure 4:**
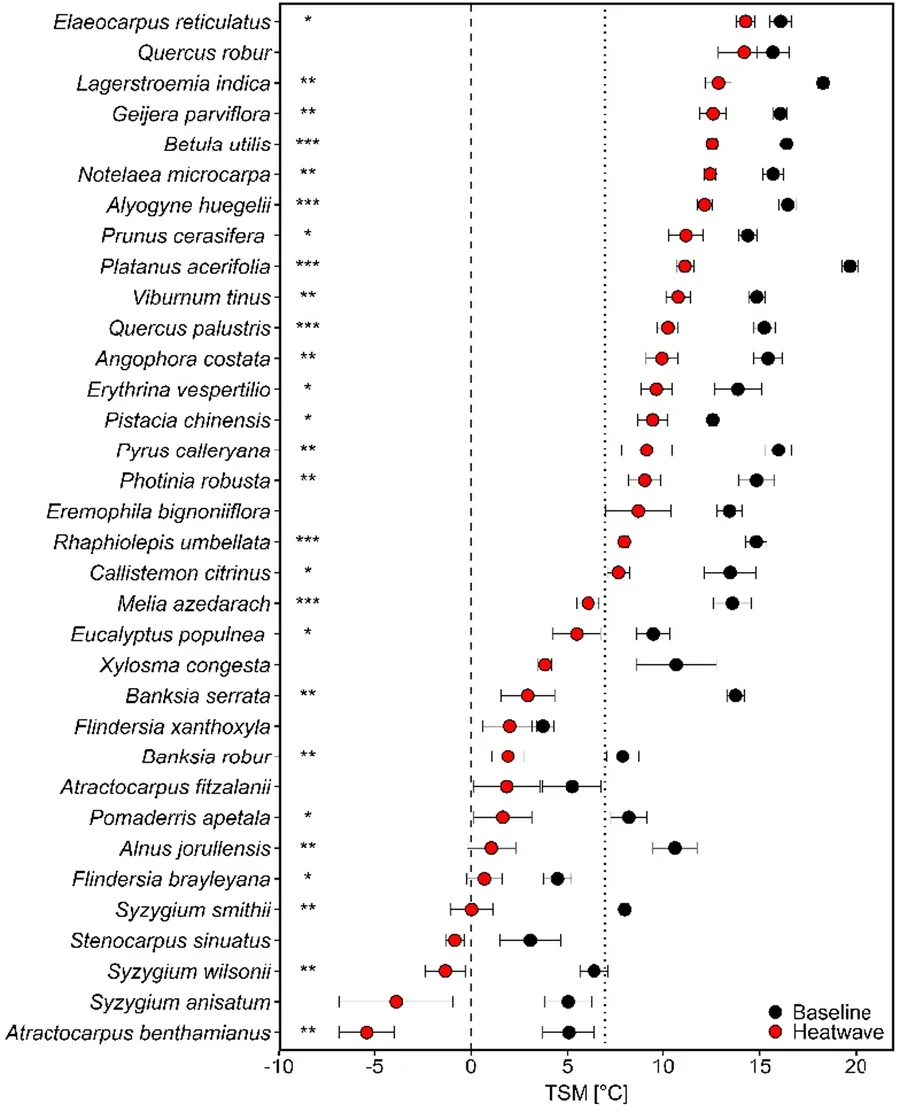
Thermal safety margin (*TSM*) under baseline (black) and +7°C heatwave (red) conditions calculated as the difference between maximum leaf temperature (*T*_*leaf_max*_) and heat tolerance (*T*_*crit*_) for 34 tree or shrub species. The species are rank‐ordered according to their mean heatwave *TSM* value (*n* = 3–5 plants per species). The dashed black line indicates a zero *TSM* value, and the dotted grey line at *TSM* of 7°C illustrates the size of the increase in peak daily air temperature between baseline and heatwave conditions. Error bars represent SE. Asterisks denote acclimation of *T*_*crit*_ during the heatwave according to Student's *t*‐tests: ***p < 0.001; **p < 0.01; *p < 0.05. [Color figure can be viewed at wileyonlinelibrary.com]

*TSMs* decreased during the heatwave (*F*_1,265_ = 652.70, *p* < 0.001), with baseline and heatwave *TSM* being positively correlated across species (*ρ* = 0.91, *p* < 0.001). The magnitude of the decline in *TSM* during the heatwave depended on species (treatment × species: *F*_1,30_ = 4.13, *p* < 0.001). Mean *TSM* differed among species by 19.7°C under heatwave conditions (*F*_1,30_ = 41.71, *p* < 0.001), ranging from −5.4°C to 14.3°C (Figure 4, Table S4), owing to the wide range of *T*_*leaf_max*_. Under baseline conditions, seven species had mean *TSM* lower than 7°C (Figure 4, Table S4), making them potentially prone to damage in the +7°C heatwave treatment. Indeed, five of these seven species had mean *TSM* < 1°C under heatwave conditions (Figure 4, Table S4). Three of these seven species exhibited no acclimation in *T*_*crit*_ after the heatwave (*Flindersia brayleyana, S. anisatum, S. wilsonii*), while mean *T*_*crit*_ significantly decreased by −2.8°C in *A. benthamianus* (Table S4). The other three species (*Atractocarpus fitzalanii*, *Flindersia xanthoxyla*, *Stenocarpus sinuatus*) did show acclimation in mean *T*_*crit*_ by +2°–3°C during the heatwave, although this was insufficient to prevent negative *TSMs* for *S. sinuatus*, due to its higher *T*_*leaf_max*_ (Table S4). The two species with positive *TSMs* (~2°C) during the heatwave were *A. fitzalanii* and *F. xanthoxyla*, owing to acclimation in *T*_crit_ and moderate increases in *T*_*leaf_max*_ of ~5°C (i.e., 2°C below the +7°C heatwave treatment; Table S4).

**Figure 5:**
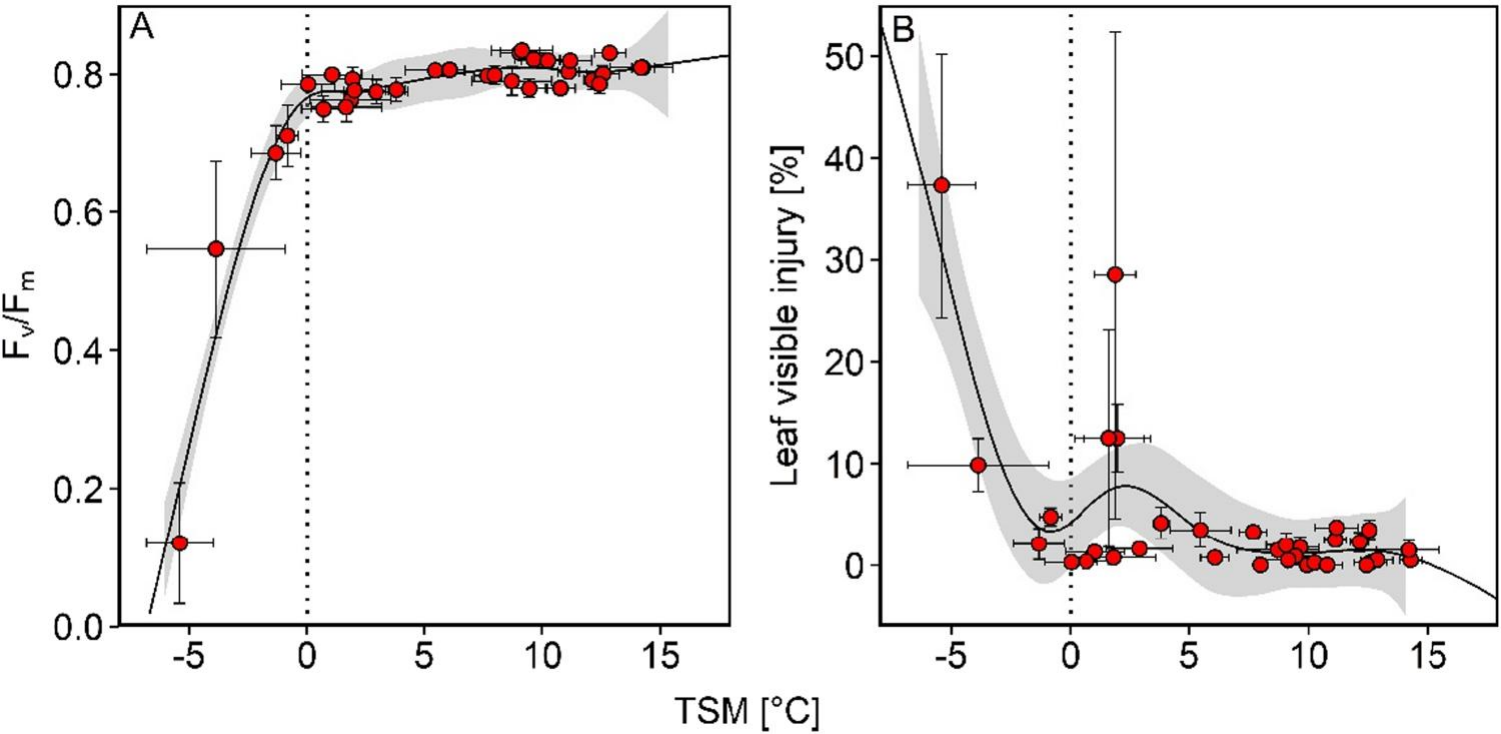
(A) Maximum quantum yield of PS II (*F*_*v*_ */F*_*m*_) and (B) leaf visible injury were nonlinearly related to thermal safety margin (*TSM*) under +7°C heatwave conditions in 34 tree or shrub species. In (A), a generalised additive model of heatwave *F*_*v*_ */F*_*m*_ and heatwave *TSM* was used (*F*_5.9,7_ = 59.92, *r* ^2^ = 0.93, *p* < 0.001). In (B), a generalised additive model of leaf visible injury and *TSM* was used (*F*_1.4,1.8_ = 6.87, *r* ^2^ = 0.31, *p* < 0.001). Values are species means (*n* = 3–5 plants per species); error bars represent SE. Vertical dotted lines indicate the zero *TSM* value. [Color figure can be viewed at wileyonlinelibrary.com]

**Figure 6:**
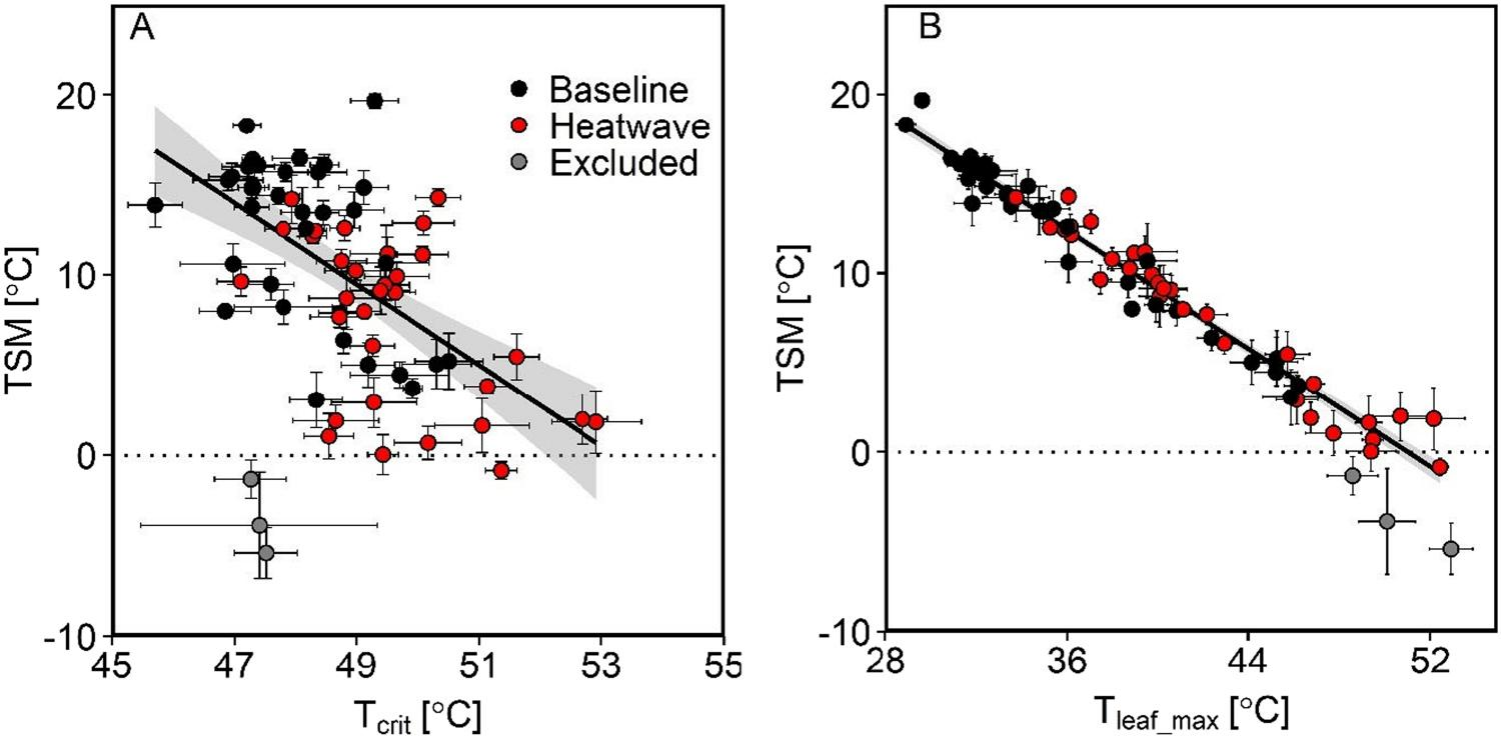
Relationships between thermal safety margin (*TSM*) and its components: (A) leaf critical temperature (*T*_*crit*_) and (B) maximum leaf temperature (*T*_*leaf_max*_). The fitted linear relationships: *TSM* = 119.63 – 2.25 × *T*_*crit*_ (*r* ^2^ = 0.38, *p* < 0.001, *n* = 65) and *TSM* = 42.09 – 0.82 × *T*_*leaf_max*_ (*r* ^2^ = 0.97, *p* < 0.001, *n* = 65). Values are species means (*n* = 3–5 plants per species); error bars represent SE. Horizontal dotted lines indicate the zero *TSM* value. The three grey points were excluded from analysis, owing to heat damage to *Atractocarpus benthamianus*, *Syzygium anisatum* and *Syzygium wilsonii*. [Color figure can be viewed at wileyonlinelibrary.com]

As species' *TSM* fell below zero, their *F*_*v*_ */F*_*m*_ declined (*F*_5.9,7_ = 59.92, *r* ^2^ = 0.93, *p* < 0.001, *n* = 34; Figure 5A) and visible injury increased nonlinearly under heatwave conditions (*F*_1.4,1.8_ = 6.87, *r* ^2^ = 0.31, *p* = 0.003; Figure 5B). *F*_*v*_ */F*_*m*_ and visible injury were negatively but not strongly correlated (*r* = −0.47, *p* = 0.005). Species' *F*_*v*_ */F*_*m*_ (*F*_3,3.7_ = 6.49, *r* ^2^ = 0.41, *p* = 0.001, *n* = 34; Figure S3A) and visible injury under heatwave conditions (*F*_1,1_ = 9.18, *r* ^2^ = 0.20, *p* = 0.005; Figure S3B) were also nonlinearly correlated to their *T*_*leaf_max*_, although the relationships were weaker. Mean *TSM* across all species was more strongly correlated to species' *T*_*leaf_max*_ (*r* ^2^ = 0.97, *p* < 0.001, *n* = 65) than their *T*_*crit*_ (*r* ^2^ = 0.38, *p* < 0.001, *n* = 65; Figure 6).

## 4 Discussion

We implemented an experimental 6‐day +7°C heatwave with a peak air temperature of 41°C, finding that diverse tree and shrub species generally increased *T*_*crit*_ (Figure 1), with Δ*T*_*crit*_ ranging from 1 to 4°C (Figure S2, Table S4). Species differences in Δ*T*_*crit*_ were not related to phylogeny or climate zone. While many species showed short‐term acclimation of *T*_*crit*_ to high temperature exposure (20 of 48 species, 42%), other species did not acclimate (27 species, 56%) or were impaired by the heat stress, resulting in decreased *T*_*crit*_ after the heatwave (1 species, 2%; Figure 1). Peak maximum leaf temperature (*T*_*leaf_max*_) was a key correlate of *T*_*crit*_ differences among plant species (Figure 2A) and of the plasticity of *T*_*crit*_ among species (Figure 3). Species with high Δ*T*_*crit*_ also had higher Δ*T*_*leaf_max*_, with leaves being 7°‒12°C hotter during the heatwave than under baseline conditions (Figure 3). We also found that *TSM* was a robust predictor of heat damage across diverse tree and shrub species from contrasting climate zones (Figures 4 and 5). Species differences in *TSMs* were strongly driven by *T*_*leaf_max*_ rather than *T*_*crit*_ (Figure 6), and *T*_*leaf_max*_ was also able to predict species vulnerability to heatwaves (Figure S3). Taken together, our results suggest that *T*_*leaf_max*_ alone is more informative than *T*_*crit*_ for ranking species heat tolerance, but *TSMs* remain valuable for identifying which species are most vulnerable to heatwaves.

### 4.1 Species Vary in Their Ability to Acclimate to Heat Stress

Prior studies have documented latitudinal, climatic and phylogenetic patterns in heat tolerance among plant species, finding that species from warmer places of origin have higher heat tolerance than species with cooler origins (Knight and Ackerly 2002; Lancaster and Humphreys 2020; O'sullivan et al. 2017; Perez and Feeley 2020; Zhu et al. 2018). Here, we expected diverse tree and shrub species to acclimate to heatwave exposure through induced heat tolerance. Our first hypothesis was generally supported: *T*_*crit*_ was plastic, exhibiting short‐term thermal acclimation to heatwave exposure (Figure 1), with a mean increase of +1.2°C across species (Figure S2). This means an increase in *T*_*crit*_ is equivalent to an acclimation of 0.20°C per 1°C increase in air temperature, assuming a linear response to air temperature. This magnitude of change is similar to the *T*_*crit*_ increase of ~0.25°C per 1°C increase in air temperature found in previous experiments with heatwave treatments (Drake et al. 2018; Zhu et al. 2018). Plant heat tolerance has recently been shown to acclimate quickly to changes in leaf temperature, with increases in *T*_*crit*_ occurring within 2 h and lasting for several days (Bison and Michaletz 2024; Zhu et al. 2024). Our findings support the conclusion that most trees and shrubs can mitigate heat stress within days by rapidly acclimating to high temperature exposure through increased *T*_*crit*_.

Acclimation of *T*_*crit*_ was not found for all study species, however, suggesting there are thermal limits for plant acclimation to heat stress, which vary across species. There were 28 species with no significant change or a decrease in *T*_*crit*_ (58%; Figure 1, Table S4). Short‐term acclimation to high temperature during heatwaves is metabolically complex and costly (Aspinwall et al. 2019), such as adjustment of membrane lipid composition (Hochachka and Somero 2002; Zhu et al. 2018), production of heat shock proteins (Vierling 1991), and/or the accumulation of protectant osmolytes (Hüve et al. 2006). For some species, there was either a lack of resources or there was no need to increase their *T*_*crit*_, perhaps because 41°C was not high enough to trigger any response. The 41°C heatwave did surpass the upper temperature threshold for at least three species (*A. benthamianus*, *S. anisatum*, *S. wilsonii*), however. These species had no thermal acclimation of *T*_*crit*_ plus damage to their photosynthetic apparatus (Figure 5A) and visible heat damage to leaves (Figure 5B). All three species are understory rainforest species from the (sub)tropics, which may be particularly vulnerable to heatwaves when grown under high irradiance. Our results suggest that failure to thermally acclimate to changing environmental conditions can constitute a major threat to plant growth and survival.

We further tested if induced heat tolerance, through short‐term acclimation to heatwave exposure, had any climatic or phylogenetic dependence across species. Among 47 diverse tree and shrub species from 25 families originating from temperate to tropical climate zones, we found that neither the native climate of origin nor evolutionary history was a determinant of tree and shrub species differences in Δ*T*_*crit*_. Previous studies have reported either a lack of relationship (Bison and Michaletz 2024) or weak relationships between constitutive heat tolerance and phylogeny (Lancaster and Humphreys 2020; Perez and Feeley 2021; Slot et al. 2021), as well as an association of *T*_*crit*_ and climate of origin (Zhu et al. 2018) but not Δ*T*_*crit*_ and biome of origin (Harris et al. 2024). Taken together, the plasticity of heat tolerance (Δ*T*_*crit*_) is not likely to be strongly shaped by evolutionary ancestry or climate of origin (Harris et al. 2024), unlike constitutive heat tolerance (Knight and Ackerly 2002; Knight and Ackerly 2003; Lancaster and Humphreys 2020).

### 4.2 *T*_*leaf_max*_ Was a Key Correlate of *T*_*crit*_ Variation Among Species

Maximum leaf temperature (*T*_*leaf_max*_) serves as an integrative trait as it is influenced by numerous physical and physiological leaf traits that affect leaf energy balance (Curtis et al. 2012; Leigh et al. 2012). Peak *T*_*leaf_max*_ is related to leaf size, thickness, reflectance and angle (Curtis et al. 2012; Leigh et al. 2012; Leigh et al. 2017), as well as leaf physiological traits that are dynamically regulated, such as the effectiveness of transpiration in regulating leaf temperature during heatwaves (Drake et al. 2018; Gates 1980; Marchin et al. 2022). We found that *T*_*crit*_ exhibited a common linear relationship with *T*_*leaf_max*_ and *T*_*diff*_ across species in both non‐heatwave and heatwave conditions (Figure 2), in support of our second hypothesis. Perez and Feeley (2020) did not find a correlation between *T*_*leaf_max*_ and *T*_*crit*_ for 19 tropical plant species measured in a common garden, although there was a correlation between *T*_*leaf_max*_ and *T*_50_, a different metric of photosynthetic heat tolerance. All our study plants were well‐watered and measured in a controlled glasshouse environment, so *T*_*leaf_max*_ was not confounded by any difference in plant water availability, as is common under field conditions. We did observe higher maximum *T*_*leaf_max*_ and *T*_*diff*_ in the HIE glasshouse than in the MQ glasshouse, with increases of ~10°C (Figure S1), likely due to differences in maximum irradiance between the two glasshouse environments. Despite these environmental differences, *T*_*leaf_max*_ and *T*_*diff*_ had consistent and common relationships with *T*_*crit*_ in response to heatwave treatment both within and across glasshouse locations when combined (Figure 2). Maximum leaf temperature (*T*_*leaf_max*_) explained a large portion (~55%) of the variation in species *T*_*crit*_ in our study (Figure 2A), suggesting that regulation of peak leaf temperature is an important mechanism for preventing heat damage in plants.

Regulation of transpiration is highly effective at preventing leaves from overheating during heatwaves, particularly for plants with water access (Marchin et al. 2023). Given higher atmospheric vapour pressure deficits during heatwaves are often associated with stomatal closure and reduced transpiration (Grossiord et al. 2020), it could be expected that *T*_*diff*_ would increase during our heatwave, but we found no such trend here (Figure 2B). Instead, our species had adequate water in a humid glasshouse and were able to maintain or increase transpiration to cool their leaves during the heatwave (Marchin et al. 2022). Species differences in mean *T*_*leaf_max*_ spanned a large range (34°‒53°C), about 19°C under heatwave conditions, from −7°C cooler to +12°C warmer than peak air temperature (Table S4). The species with low Δ*T*_*leaf_max*_, or leaves that remained a mild 2°‒6°C hotter during the +7°C heatwave, relative to baseline conditions, had little acclimation of *T*_*crit*_ (Figure 3). Conversely, species with high Δ*T*_*leaf_max*_, i.e., leaves that were 7°‒12°C hotter during the heatwave than under baseline conditions, had high plasticity of *T*_*crit*_ (i.e., Δ*T*_*crit*_ > 1.5°C; Figure 3). Despite several notable exceptions (i.e., species with high Δ*T*_*leaf_max*_ but low Δ*T*_*crit*_), this nonlinear relationship suggests that species utilised different physiological mechanisms – either large acclimation of *T*_*crit*_, or large increase in transpiration, but not both simultaneously – to survive heatwave events.

We found strong evidence that peak leaf temperature is associated with species differences in *T*_*crit*_ (Figure 3) and should be considered a major driver of short‐term thermal acclimation of *T*_*crit*_ during heatwaves. The relationships between *T*_*crit*_ and *T*_*leaf_max*_ reported here (Figures 2 and 3), if widespread among species, could partly explain observed *T*_*crit*_ and *T*_*crit*_ acclimation differences among species both within and across sites (Curtis et al. 2014; Knight and Ackerly 2002; Leon‐Garcia and Lasso 2019; O'Sullivan et al. 2017; Sastry and Barua 2017). Compared to *T*_*crit*_, which is time‐consuming to measure, *T*_*leaf_max*_ can provide an alternative measure of heatwave vulnerability among species that is more rapid and efficient. Being able to rapidly screen plants for their vulnerability to heatwaves in situ would aid in predicting plant responses and informing the selection of heat‐tolerant plants to mitigate the impact of climate change in managed environments and cities (Ossola and Lin 2021).

### 4.3 TSMs as Predictors of Heatwave Vulnerability Among Species

Various definitions of *TSM* have been used, but those based on leaf, rather than air, temperature are more useful for determining vulnerability to heat stress in plants (Cook et al. 2021). Here, we found that *TSMs* based on leaf temperature could reliably assess species vulnerability to heatwaves (Figure 5), in support of our third hypothesis. Under heatwave conditions, species with lower *TSMs* were more visibly damaged (Figure 5B) and had higher photoinhibition during the heatwave (Figure 5A) than species with higher *TSMs*. Similarly, populations of *Corymbia calophylla* originating from hotter climates were previously found to be more heat tolerant than populations from cooler climates, due to their greater *TSMs* and reduced damage after an experimental heatwave (Ahrens et al. 2021). Yet, it has not been clear if pre‐heatwave *TSMs* could effectively assess species vulnerability to heatwaves. We found that *TSMs* under non‐heatwave conditions are a reliable predictor of species vulnerability to heat – the seven species with vulnerably low *TSMs* (i.e., < 7°C in baseline conditions) had negative or low *TSMs* during the heatwave (< 2°C; Figure 4). Heat‐tolerant species can be identified by their comparatively higher *TSMs*, at least when species are grown and compared in a common environment.

It is informative to consider how the two components of *TSM* – *T*_*crit*_ and *T*_*leaf_max*_ – each contributed to influencing *TSM* across species. We found that species' *TSMs* were more strongly correlated to their *T*_*leaf_max*_ than their *T*_*crit*_ (Figure 6). This may be partly due to the larger range in heatwave *T*_*leaf_max*_ (19°C; 34°‒53°C) than in *T*_*crit*_ (7°C; 46°‒53°C) across the species in our study. Interestingly, it suggests that regulation of *T*_*leaf_max*_ may be more important than changes in *T*_*crit*_ in determining *TSM* during heatwaves. Indeed, species' *T*_*leaf_max*_ was also correlated to their vulnerability to heatwaves (Figure S3). The 10 species with the hottest leaves during the heatwave (mean *T*_*leaf_max*_ > 47°C) all had low *TSM* (≤ 2°C) and were at risk for damage, whereas species with cooler leaves (mean *T*_*leaf_max*_ < 45°C) maintained higher *TSM* (> 5°C; Table S4) and avoided heat damage (Figure S3). Our findings highlight the importance of measuring *T*_*leaf_max*_, in addition to or instead of *T*_*crit*_, to assess species vulnerability to heatwaves.

### 4.4 Limitations and Ways Forward

A major challenge in plant ecophysiology is to reliably identify heat‐tolerant tree and shrub species that can be used for urban planting and forest restoration projects in our changing climate. Many studies, including our current study, have focused on quantifying a single temperature threshold, such as *T*_*crit*_ or *T*_*50*_, to rank species thermal tolerance. We found that species' heat tolerance rankings based on *T*_*crit*_ were not consistent across measurement temperatures (Figure 1). Critical limits such as *T*_*crit*_ ignore that physiological stress is cumulative in nature and depends on both the intensity and duration of heat exposure (Cook et al. 2024; Neuner and Buchner 2023). For plants, the severity of thermal damage is also dependent on the presence of other interacting stresses, such as low plant water availability (Marchin et al. 2022; Teskey et al. 2015) or high irradiance. Plant heat tolerance is not a static trait but is highly dynamic, acclimating quickly to changes in leaf microclimate over short timescales (Bison and Michaletz 2024; Zhu et al. 2024). Thus, despite the increasing number of studies reporting critical thermal thresholds for plant species, it is difficult to compare species across studies that have different growth conditions, treatments and measurement methods (Geange et al. 2021).

Our study has identified two alternative parameters to critical limits, such as *T*_*crit*_, which could potentially aid in more reliably ranking species' heat tolerance – *T*_*leaf_max*_ and *TSM*. As mentioned previously, *T*_*leaf_max*_ has several advantages over *T*_*crit*_; its measurement is more rapid, requires less expensive equipment, and was found here to be closely correlated to both *T*_*crit*_ (Figure 2A) and, most critically, heat damage across species (Figure S3). One disadvantage is that the temperature threshold for damage differs across species (Figure 1) due to differences in leaf physical and physiological traits, so it is difficult to split tolerant from vulnerable species based on *T*_*leaf_max*_ measurement alone. For example, we observed that only three understory rainforest species were notably damaged (*A. benthamianus*, *S. anisatum*, *S. wilsonii*), having heatwave *F*_*v*_ */F*_*m*_ < 0.7 (Figure S3), even though another seven species also had mean *T*_*leaf_max*_ above 47°C (Table S4).

We can therefore conclude that *TSMs* are the most reliable parameter to assess species vulnerability to heatwaves. They were stable across measurement temperatures (Figure 4), had a clear threshold (i.e., zero) for damage across species due to integration of *T*_*crit*_ with *T*_*leaf_max*_, and were correlated with heat damage across species (Figure 5). Heatwave *TSM* correctly identified the three most vulnerable species (*A. benthamianus*, *S. anisatum*, *S. wilsonii*; Figure 4), which were actually ranked closer to the tolerant end of the spectrum based on their baseline *T*_*crit*_ (Figure 1). It appears that species with higher *T*_*leaf_max*_ may require higher *T*_*crit*_ to survive heatwaves, leading to narrow *TSM* as temperatures approach the limits for plant function. This supports the idea that species with higher heat tolerance are operating closer to the upper thermal threshold for plants (Slot et al. 2021) and may be more susceptible to thermal damage under extreme conditions (Perez and Feeley 2020). If so, *T*_*leaf_max*_, rather than *T*_*crit*_, is largely responsible for the variation in *TSMs* across species (Figure 6), so its measurement, in addition to or instead of *T*_*crit*_, is needed to assess species vulnerability to heatwaves. A deeper knowledge of differences in *TSMs* among a larger set of plant species would critically improve current predictions and models of the impact of heatwaves on plants in a changing climate.

## Supporting information

Supplementary data.

## Data Availability

The data that support the findings of this study are openly available in Figshare at https://doi.org/10.6084/m9.figshare.29345549.v1.

## References

[pce70041-bib-0001] Ahrens, C. W. , A. Challis , M. Byrne , et al. 2021. “Repeated Extreme Heatwaves Result in Higher Leaf Thermal Tolerances and Greater Safety Margins.” New Phytologist 232: 1212–1225. 34292598 10.1111/nph.17640

[pce70041-bib-0002] Allakhverdiev, S. I. , Y. M. Feyziev , A. Ahmed , et al. 1996. “Stabilization of Oxygen Evolution and Primary Electron Transport Reactions in Photosystem II Against Heat Stress With Glycinebetaine and Sucrose.” Journal of Photochemistry and Photobiology, B: Biology 34: 149–157. 22872909 10.1016/1011-1344(95)07276-4

[pce70041-bib-0003] Aspinwall, M. J. , S. Pfautsch , M. G. Tjoelker , et al. 2019. “Range Size and Growth Temperature Influence *Eucalyptus* Species Responses to an Experimental Heatwave.” Global Change Biology 25: 1665–1684. 30746837 10.1111/gcb.14590

[pce70041-bib-0004] Bilger, H.‐W. , U. Schreiber , and O. L. Lange . 1984. “Determination of Leaf Heat Resistance: Comparative Investigation of Chlorophyll Fluorescence Changes and Tissue Necrosis Methods.” Oecologia 63: 256–262. 28311022 10.1007/BF00379886

[pce70041-bib-0005] Bison, N. N. , and S. T. Michaletz . 2024. “Variation in Leaf Carbon Economics, Energy Balance, and Heat Tolerance Traits Highlights Differing Timescales of Adaptation and Acclimation.” New Phytologist 242: 1919–1931. 38532535 10.1111/nph.19702

[pce70041-bib-0006] Bita, C. E. , and T. Gerats . 2013. “Plant Tolerance to High Temperature in a Changing Environment: Scientific Fundamentals and Production of Heat Stress‐Tolerant Crops.” Frontiers in Plant Science 4: 273. 10.3389/fpls.2013.00273. 23914193 PMC3728475

[pce70041-bib-0007] Blomberg, S. P. , and T. Garland . 2002. “Tempo and Mode in Evolution: Phylogenetic Inertia, Adaptation and Comparative Methods.” Journal of Evolutionary Biology 15: 899–910.

[pce70041-bib-0008] Breshears, D. D. , J. B. Fontaine , K. X. Ruthrof , et al. 2021. “Underappreciated Plant Vulnerabilities to Heat Waves.” New Phytologist 231: 32–39. 33728638 10.1111/nph.17348

[pce70041-bib-0009] Bukhov, N. G. , C. Wiese , S. Neimanis , and U. Heber . 1999. “Heat Sensitivity of Chloroplasts and Leaves: Leakage of Protons From Thylakoids and Reversible Activation of Cyclic Electron Transport.” Photosynthesis Research 59: 81–93.

[pce70041-bib-0010] Burley, H. , L. J. Beaumont , A. Ossola , et al. 2019. “Substantial Declines in Urban Tree Habitat Predicted Under Climate Change.” Science of the Total Environment 685: 451–462. 31176230 10.1016/j.scitotenv.2019.05.287

[pce70041-bib-0011] Chamberla, S. , and E. Szocs . 2013. Taxize – Taxonomic Search and Retrieval in R. F1000Research. 10.12688/f1000research.2-191.v1 PMC3901538 24555091

[pce70041-bib-0012] Chambers, J. , and T. Hastie . 1992. Statistical Models in S. Routledge.

[pce70041-bib-0013] Cook, A. M. , N. Berry , K. V. Milner , and A. Leigh . 2021. “Water Availability Influences Thermal Safety Margins for Leaves.” Functional Ecology 35: 2179–2189.

[pce70041-bib-0014] Cook, A. M. , E. L. Rezende , K. Petrou , and A. Leigh . 2024. “Beyond a Single Temperature Threshold: Applying a Cumulative Thermal Stress Framework to Plant Heat Tolerance.” Ecology Letters 27: e14416. 38549256 10.1111/ele.14416

[pce70041-bib-0015] Cunningham, S. C. , and J. Read . 2006. “Foliar Temperature Tolerance of Temperate and Tropical Evergreen Rain Forest Trees of Australia.” Tree Physiology 26: 1435–1443. 16877328 10.1093/treephys/26.11.1435

[pce70041-bib-0016] Curtis, E. M. , C. A. Knight , K. Petrou , and A. Leigh . 2014. “A Comparative Analysis of Photosynthetic Recovery From Thermal Stress: A Desert Plant Case Study.” Oecologia 175: 1051–1061. 24958368 10.1007/s00442-014-2988-5

[pce70041-bib-0017] Curtis, E. M. , A. Leigh , and S. Rayburg . 2012. “Relationships Among Leaf Traits of Australian Arid Zone Plants: Alternative Modes of Thermal Protection.” Australian Journal of Botany 60: 471–483.

[pce70041-bib-0018] Doughty, C. E. , J. M. Keany , B. C. Wiebe , et al. 2023. “Tropical Forests Are Approaching Critical Temperature Thresholds.” Nature 621: 105–111. 37612501 10.1038/s41586-023-06391-z

[pce70041-bib-0019] Drake, J. E. , M. G. Tjoelker , A. Vårhammar , et al. 2018. “Trees Tolerate an Extreme Heatwave via Sustained Transpirational Cooling and Increased Leaf Thermal Tolerance.” Global Change Biology 24: 2390–2402. 29316093 10.1111/gcb.14037

[pce70041-bib-0020] Fox, J. , and S. Weisberg . 2019. An R Companion to Applied Regression. Sage.

[pce70041-bib-0021] Gates, D. M. 1980. Biophysical Ecology. Springer‐Verlag.

[pce70041-bib-0022] GBIF . 2019. Global Biodiversity Information Facility.

[pce70041-bib-0023] Geange, S. R. , P. A. Arnold , A. A. Catling , et al. 2021. “The Thermal Tolerance of Photosynthetic Tissues: A Global Systematic Review and Agenda for Future Research.” New Phytologist 229: 2497–2513. 33124040 10.1111/nph.17052

[pce70041-bib-0024] Grossiord, C. , T. N. Buckley , L. A. Cernusak , et al. 2020. “Plant Responses to Rising Vapor Pressure Deficit.” New Phytologist 226: 1550–1566. 32064613 10.1111/nph.16485

[pce70041-bib-0025] Guilioni, L. 1997. “Heat Stress‐Induced Abortion of Buds and Flowers in Pea: Is Sensitivity Linked to Organ Age or to Relations Between Reproductive Organs?.” Annals of Botany 80: 159–168.

[pce70041-bib-0026] Hammond, W. M. , A. P. Williams , J. T. Abatzoglou , et al. 2022. “Global Field Observations of Tree Die‐Off Reveal Hotter‐Drought Fingerprint for Earth's Forests.” Nature Communications 13: 1761. 10.1038/s41467-022-29289-2 PMC8983702 35383157

[pce70041-bib-0027] Harris, R. J. , P. R. Alvarez , C. Bryant , et al. 2024. “Acclimation of Thermal Tolerance in Juvenile Plants From Three Biomes Is Suppressed When Extremes Co‐Occur.” Conservation Physiology 12, no. 1: coae027. 39850455 10.1093/conphys/coae027 PMC11756708

[pce70041-bib-0028] Havaux, M. 1993. “Rapid Photosynthetic Adaptation to Heat‐Stress Triggered in Potato Leaves by Moderately Elevated‐Temperatures.” Plant, Cell & Environment 16: 461–467.

[pce70041-bib-0029] Havaux, M. , and F. Tardy . 1996. “Temperature‐Dependent Adjustment of the Thermal Stability of Photosystem II In Vivo: Possible Involvement of Xanthophyll‐Cycle Pigments.” Planta 198: 324–333.

[pce70041-bib-0030] He, M. , C.‐Q. He , and N.‐Z. Ding . 2018. “Abiotic Stresses: General Defenses of Land Plants and Chances for Engineering Multistress Tolerance.” Frontiers in Plant Science 9: 1771. 10.3389/fpls.2018.01771. 30581446 PMC6292871

[pce70041-bib-0031] Hochachka, P. W. , and G. N. Somero . 2002. Biochemical Adaptation: Mechanism and Process in Physiological Evolution. Oxford University Press.

[pce70041-bib-0032] Hüve, K. , I. Bichele , M. Tobias , and U. Niinemets . 2006. “Heat Sensitivity of Photosynthetic Electron Transport Varies During the Day Due to Changes in Sugars and Osmotic Potential.” Plant, Cell & Environment 29: 212–228. 10.1111/j.1365-3040.2005.01414.x 17080637

[pce70041-bib-0033] IPCC. 2022. “Summary for Policymakers.” In Climate Change 2022: Impacts, Adaptation, and Vulnerability. Contribution of Working Group II to the Sixth Assessment Report of the Intergovernmental Panel on Climate Change, edited by H.‐O. Pörtner , D. C. Roberts and E. S. Poloczanska , et al., 3–33. Cambridge University Press.

[pce70041-bib-0034] KBD . 2020. The International Plant Names Index and World Checklist of Selected Plant Families 2020.

[pce70041-bib-0035] Kim, J. , and M. J. Sanderson . 2008. “Penalized Likelihood Phylogenetic Inference: Bridging the Parsimony‐Likelihood Gap.” Systematic Biology 57: 665–674. 18853355 10.1080/10635150802422274

[pce70041-bib-0036] Kitudom, N. , S. Fauset , Y. Zhou , et al. 2022. “Thermal Safety Margins of Plant Leaves Across Biomes Under a Heatwave.” Science of the Total Environment 806: 150416. 34852425 10.1016/j.scitotenv.2021.150416

[pce70041-bib-0037] Knight, C. A. , and D. D. Ackerly . 2002. “An Ecological and Evolutionary Analysis of Photosynthetic Thermotolerance Using the Temperature‐Dependent Increase in Fluorescence.” Oecologia 130: 505–514. 28547251 10.1007/s00442-001-0841-0

[pce70041-bib-0038] Knight, C. A. , and D. D. Ackerly . 2003. “Evolution and Plasticity of Photosynthetic Thermal Tolerance, Specific Leaf Area and Leaf Size: Congeneric Species From Desert and Coastal Environments.” New Phytologist 160: 337–347. 33832168 10.1046/j.1469-8137.2003.00880.x

[pce70041-bib-0039] Krause, G. H. , A. W. Cheesman , K. Winter , B. Krause , and A. Virgo . 2013. “Thermal Tolerance, Net CO_2_ Exchange and Growth of a Tropical Tree Species, Ficus Insipida, Cultivated at Elevated Daytime and Nighttime Temperatures.” Journal of Plant Physiology 170: 822–827. 23399405 10.1016/j.jplph.2013.01.005

[pce70041-bib-0040] Krause, G. H. , and E. Weis . 1991. “Chlorophyll Fluorescence and Photosynthesis – The Basics.” Annual Review of Plant Physiology and Plant Molecular Biology 42: 313–349.

[pce70041-bib-0041] Krause, G. H. , K. Winter , B. Krause , et al. 2010. “High‐Temperature Tolerance of a Tropical Tree, Ficus Insipida: Methodological Reassessment and Climate Change Considerations.” Functional Plant Biology 37: 890–900.

[pce70041-bib-0042] Kullberg, A. T. , and K. J. Feeley . 2024. “Seasonal Acclimation of Photosynthetic Thermal Tolerances in Six Woody Tropical Species Along a Thermal Gradient.” Functional Ecology 38: 2493–2505.

[pce70041-bib-0043] Lancaster, L. T. , and A. M. Humphreys . 2020. “Global Variation in the Thermal Tolerances of Plants.” Proceedings of the National Academy of Sciences 117: 13580–13587. 10.1073/pnas.1918162117 PMC7306813 32482870

[pce70041-bib-0044] Leigh, A. , S. Sevanto , M. C. Ball , et al. 2012. “Do Thick Leaves Avoid Thermal Damage in Critically Low Wind Speeds?.” New Phytologist 194: 477–487. 22296328 10.1111/j.1469-8137.2012.04058.x

[pce70041-bib-0045] Leigh, A. , S. Sevanto , J. D. Close , and A. B. Nicotra . 2017. “The Influence of Leaf Size and Shape on Leaf Thermal Dynamics: Does Theory Hold Up Under Natural Conditions?.” Plant, Cell & Environment 40: 237–248. 10.1111/pce.12857 28026874

[pce70041-bib-0046] Leon‐Garcia, I. V. , and E. Lasso . 2019. “High Heat Tolerance in Plants From the Andean Highlands: Implications for Paramos in a Warmer World.” PLoS One 14: e0224218. 31693675 10.1371/journal.pone.0224218 PMC6834248

[pce70041-bib-0047] Mangiafico, S. S. 2016. Summary and Analysis of Extension Program Evaluation in R. Version 1.18.1.

[pce70041-bib-0048] Manzi, O. J. L. , M. Wittemann , M. E. Dusenge , et al. 2024. “Canopy Temperatures Strongly Overestimate Leaf Thermal Safety Margins of Tropical Trees.” New Phytologist 243: 2115–2129. 39073111 10.1111/nph.20013

[pce70041-bib-0049] Marchin, R. M. , D. Backes , A. Ossola , M. R. Leishman , M. G. Tjoelker , and D. S. Ellsworth . 2022. “Extreme Heat Increases Stomatal Conductance and Drought‐Induced Mortality Risk in Vulnerable Plant Species.” Global Change Biology 28: 1133–1146. 34741566 10.1111/gcb.15976 PMC9299030

[pce70041-bib-0050] Marchin, R. M. , B. E. Medlyn , M. G. Tjoelker , and D. S. Ellsworth . 2023. “Decoupling Between Stomatal Conductance and Photosynthesis Occurs Under Extreme Heat in Broadleaf Tree Species Regardless of Water Access.” Global Change Biology 29: 6319–6335. 37698501 10.1111/gcb.16929

[pce70041-bib-0051] Maxwell, K. , and G. N. Johnson . 2000. “Chlorophyll Fluorescence – A Practical Guide.” Journal of Experimental Botany 51: 659–668. 10938857 10.1093/jxb/51.345.659

[pce70041-bib-0053] Neuner, G. , and O. Buchner . 2023. “The Dose Makes the Poison: The Longer the Heat Lasts, the Lower the Temperature for Functional Impairment and Damage.” Environmental and Experimental Botany 212: 105395.

[pce70041-bib-0054] Niinemets, Ü. 2018. “When Leaves Go Over the Thermal Edge.” Plant, Cell & Environment 41: 1247–1250. 10.1111/pce.13184 29508926

[pce70041-bib-0055] O'Sullivan, O. S. , M. A. Heskel , P. B. Reich , et al. 2017. “Thermal Limits of Leaf Metabolism Across Biomes.” Global Change Biology 23: 209–223. 27562605 10.1111/gcb.13477

[pce70041-bib-0056] O'Sullivan, O. S. , K. W. L. K. Weerasinghe , J. R. Evans , J. J. G. Egerton , M. G. Tjoelker , and O. K. Atkin . 2013. “High‐Resolution Temperature Responses of Leaf Respiration in Snow Gum (*Eucalyptus pauciflora*) Reveal High‐Temperature Limits to Respiratory Function.” Plant, Cell & Environment 36: 1268–1284. 10.1111/pce.12057 23278101

[pce70041-bib-0057] Ossola, A. , and B. B. Lin . 2021. “Making Nature‐Based Solutions Climate‐Ready for the 50°C World.” Environmental Science & Policy 123: 151–159.

[pce70041-bib-0058] Perez, T. M. , and K. J. Feeley . 2020. “Photosynthetic Heat Tolerances and Extreme Leaf Temperatures.” Functional Ecology 34: 2236–2245.

[pce70041-bib-0059] Perez, T. M. , and K. J. Feeley . 2021. “Weak Phylogenetic and Climatic Signals in Plant Heat Tolerance.” Journal of Biogeography 48: 91–100.

[pce70041-bib-0060] Perkins‐Kirkpatrick, S. E. , and S. C. Lewis . 2020. “Increasing Trends in Regional Heatwaves.” Nature Communications 11: 3357. 10.1038/s41467-020-16970-7 PMC7334217 32620857

[pce70041-bib-0061] Poorter, H. , F. Fiorani , R. Pieruschka , et al. 2016. “Pampered Inside, Pestered Outside? Differences and Similarities Between Plants Growing in Controlled Conditions and in the Field.” New Phytologist 212: 838–855. 27783423 10.1111/nph.14243

[pce70041-bib-0062] POWO . 2019. Plants of the World Online. Facilitated by the Royal Botanical Gardens.

[pce70041-bib-0063] QGIS . 2019. QGIS Geographic Information System. Open Source Geospatial Foundation Project.

[pce70041-bib-0064] R Core Team . 2021. R: A Language and Environment for Statistical Computing. R Foundation for Statistical Computing.

[pce70041-bib-0065] Royston, P. 1995. “Remark as R94: A Remark on Algorithm as 181: The W‐Test for Normality.” Journal of the Royal Statistical Society. Series C (Applied Statistics). 44: 547–551.

[pce70041-bib-0066] Sage, R. F. , and D. S. Kubien . 2007. “The Temperature Response of C_3_ and C_4_ Photosynthesis.” Plant, Cell & Environment 30: 1086–1106. 10.1111/j.1365-3040.2007.01682.x 17661749

[pce70041-bib-0067] Sastry, A. , and D. Barua . 2017. “Leaf Thermotolerance in Tropical Trees From a Seasonally Dry Climate Varies Along the Slow‐Fast Resource Acquisition Spectrum.” Scientific Reports 7: 11246. 28900253 10.1038/s41598-017-11343-5 PMC5595873

[pce70041-bib-0068] Schreiber, U. , and J. A. Berry . 1977. “Heat‐Induced Changes of Chlorophyll Fluorescence in Intact Leaves Correlated With Damage of the Photosynthetic Apparatus.” Planta 136: 233–238. 24420396 10.1007/BF00385990

[pce70041-bib-0069] Seemann, J. R. , J. A. Berry , and W. J. S. Downton . 1984. “Photosynthetic Response and Adaptation to High Temperature in Desert Plants. A Comparison of Gas Exchange and Fluorescence Methods for Studies of Thermal Tolerance.” Plant Physiology 75: 364–368. 16663627 10.1104/pp.75.2.364 PMC1066913

[pce70041-bib-0070] Seemann, J. R. , W. J. S. Downton , and J. A. Berry . 1986. “Temperature and Leaf Osmotic Potential as Factors in the Acclimation of Photosynthesis to High Temperature in Desert Plants.” Plant Physiology 80: 926–930. 16664743 10.1104/pp.80.4.926 PMC1075231

[pce70041-bib-0071] Slot, M. , D. Cala , J. Aranda , A. Virgo , S. T. Michaletz , and K. Winter . 2021. “Leaf Heat Tolerance of 147 Tropical Forest Species Varies With Elevation and Leaf Functional Traits, but Not With Phylogeny.” Plant, Cell & Environment 44: 2414–2427. 10.1111/pce.14060 33817813

[pce70041-bib-0072] Takahashi, S. , and N. Murata . 2008. “How Do Environmental Stresses Accelerate Photoinhibition?.” Trends in Plant Science 13: 178–182. 18328775 10.1016/j.tplants.2008.01.005

[pce70041-bib-0073] Taub, D. R. , J. R. Seemann , and J. S. Coleman . 2000. “Growth in Elevated CO_2_ Protects Photosynthesis Against High‐Temperature Damage.” Plant, Cell & Environment 23: 649–656.

[pce70041-bib-0074] Teskey, R. , T. Wertin , I. Bauweraerts , M. Ameye , M. A. McGuire , and K. Steppe . 2015. “Responses of Tree Species to Heat Waves and Extreme Heat Events.” Plant, Cell & Environment 38: 1699–1712. 10.1111/pce.12417 25065257

[pce70041-bib-0075] Tiwari, R. , E. Gloor , W. J. A. da Cruz , et al. 2021. “Photosynthetic Quantum Efficiency in South‐Eastern Amazonian Trees May Be Already Affected by Climate Change.” Plant, Cell & Environment 44: 2428–2439. 10.1111/pce.13770 32339294

[pce70041-bib-0076] Valladares, F. , and R. W. Pearcy . 1997. “Interactions Between Water Stress, Sun‐Shade Acclimation, Heat Tolerance and Photoinhibition in the Sclerophyll *Heteromeles arbutifolia* .” Plant, Cell & Environment 20: 25–36.

[pce70041-bib-0077] Vierling, E. 1991. “The Roles of Heat‐Shock Proteins in Plants.” Annual Review of Plant Physiology and Plant Molecular Biology 42: 579–620.

[pce70041-bib-0078] Vollenweider, P. , and M. S. Günthardt‐Goerg . 2005. “Diagnosis of Abiotic and Biotic Stress Factors Using the Visible Symptoms in Foliage.” Environmental Pollution 137: 455–465. 16005758 10.1016/j.envpol.2005.01.032

[pce70041-bib-0079] Wahid, A. , S. Gelani , M. Ashraf , and M. Foolad . 2007. “Heat Tolerance in Plants: An Overview.” Environmental and Experimental Botany 61: 199–223.

[pce70041-bib-0080] Wood, S. N. , N. Pya , and B. Säfken . 2016. “Smoothing Parameter and Model Selection for General Smooth Models.” Journal of the American Statistical Association 111: 1548–1563.

[pce70041-bib-0081] Zhu, L. , K. J. Bloomfield , C. H. Hocart , et al. 2018. “Plasticity of Photosynthetic Heat Tolerance in Plants Adapted to Thermally Contrasting Biomes.” Plant, Cell & Environment 41: 1251–1262. 10.1111/pce.13133 29314047

[pce70041-bib-0082] Zhu, L. , A. P. Scafaro , E. Vierling , et al. 2024. “Heat Tolerance of a Tropical–Subtropical Rainforest Tree Species *Polyscias elegans*: Time‐Dependent Dynamic Responses of Physiological Thermostability and Biochemistry.” New Phytologist 241: 715–731. 37932881 10.1111/nph.19356

