# Supplementary material for: Thermal Safety Margins and Peak Leaf Temperatures Predict Vulnerability of Diverse Plant Species to an Experimental Heatwave
Source: Plant Cell Environ. 2025 Jun 29;49(10):7526–38. doi: 10.1111/pce.70041 (PMC13551656; doi:10.1111/pce.70041)
Supplement: Supplementary file 1 [file PCE-49-7526-s001.docx]

**Supplementary data**

**Thermal safety margins and peak leaf temperatures predict vulnerability of diverse plant species to an experimental heatwave**

Diana Cox^1*^; Renée M. Marchin^1*^; David S. Ellsworth^1^; Agnieszka Wujeska-Klause^1,2^; Alessandro Ossola^3,4^; Kristine Y. Crous^1^; Michelle R. Leishman^3^; Paul D. Rymer^1^; Mark G. Tjoelker^1^

^1^Hawkesbury Institute for the Environment, Western Sydney University, Penrith, NSW, 2751, Australia

^2^Urban Transformations Research Centre, Western Sydney University, Parramatta, NSW 2150, Australia

^3^Department of Biological Sciences, Macquarie University, North Ryde, NSW, 2109, Australia

^4^Department of Plant Sciences, University of California Davis, Davis, 95616, CA, USA

#### *Diana Cox and Renée M. Marchin should be considered as joint first authors

The following Supporting Data is available for this article:

- Tables S1-S4
- Figures S1-S3

Table S1. Family, growth habit, growth form, natural occurrence, and Köppen–Geiger climate zone of 50 tree or shrub species selected for study and grown in two glasshouses in Sydney, New South Wales, Australia: HIE refers to the Hawkesbury Institute for the Environment at Western Sydney University, and MQ refers to the Plant Growth Facility at Macquarie University. Species are ordered by glasshouse location, growth form, and family. Species in bold are those for which thermal safety margins were measured in this study.

| **Species** | **Family** | **Growth Habit** | **Growth Form** | **Natural Occurrence** | **Climate Zone** | **Glasshouse Location** |
| --- | --- | --- | --- | --- | --- | --- |
| *Acronychia oblongifolia* | Rutaceae | Evergreen | Tree | Rainforest | Temperate | HIE |
| *Agonis flexuosa* | Myrtaceae | Evergreen | Shrub | Coastal dunes, heathland | Temperate | HIE |
| ***Alnus jorullensis*** | **Betulaceae** | **Evergreen** | **Tree** | **Wet montane forest** | **Subtropical** | **HIE** |
| *Alphitonia excelsa* | Rhamnaceae | Evergreen | Tree | Eucalypt forest, savanna | Tropical | HIE |
| ***Atractocarpus benthamianus*** | **Rubiaceae** | **Evergreen** | **Tree** | **Rainforest** | **Subtropical** | **HIE** |
| ***Atractocarpus fitzalanii*** | **Rubiaceae** | **Evergreen** | **Tree** | **Rainforest** | **Tropical** | **HIE** |
| ***Banksia robur*** | **Proteaceae** | **Evergreen** | **Shrub** | **Coastal woodland** | **Temperate** | **HIE** |
| ***Banksia serrata*** | **Proteaceae** | **Evergreen** | **Tree** | **Dry sclerophyll forest** | **Temperate** | **HIE** |
| *Brachychiton populneus* | Malvaceae | Evergreen | Tree | Dry woodland | Arid | HIE |
| *Brachychiton rupestris* | Malvaceae | Deciduous | Tree | Bottletree scrub | Arid | HIE |
| ***Callistemon citrinus*** | **Myrtaceae** | **Evergreen** | **Shrub** | **Coastal woodland** | **Temperate** | **HIE** |
| *Ceratopetalum apetalum* | Cunoniaceae | Evergreen | Tree | Rainforest | Temperate | HIE |
| ***Eremophila bignoniiflora*** | **Scrophulariaceae** | **Evergreen** | **Shrub** | **Eucalypt woodland** | **Arid** | **HIE** |
| ***Eucalyptus populnea*** | **Myrtaceae** | **Evergreen** | **Tree** | **Eucalypt woodland** | **Arid** | **HIE** |
| *Eucryphia lucida* | Cunoniaceae | Evergreen | Tree | Wet forest | Temperate | HIE |
| ***Flindersia brayleyana*** | **Rutaceae** | **Evergreen** | **Tree** | **Rainforest** | **Tropical** | **HIE** |
| ***Flindersia xanthoxyla*** | **Rutaceae** | **Evergreen** | **Tree** | **Rainforest** | **Subtropical** | **HIE** |
| *Pittosporum angustifolium* | Pittosporaceae | Evergreen | Tree | Mallee woodland | Arid | HIE |
| *Pittosporum bicolor* | Pittosporaceae | Evergreen | Tree | Wet sclerophyll forest | Temperate | HIE |
| *Podocarpus elatus* | Podocarpaceae | Evergreen | Tree | Dry rainforest | Subtropical | HIE |
| ***Pomaderris apetala*** | **Rhamnaceae** | **Evergreen** | **Shrub** | **Wet sclerophyll forest** | **Temperate** | **HIE** |
| ***Stenocarpus sinuatus*** | **Proteaceae** | **Evergreen** | **Tree** | **Rainforest** | **Subtropical** | **HIE** |
| ***Syzygium anisatum*** | **Myrtaceae** | **Evergreen** | **Tree** | **Rainforest** | **Subtropical** | **HIE** |
| ***Syzygium smithii*** | **Myrtaceae** | **Evergreen** | **Tree** | **Rainforest** | **Temperate** | **HIE** |
| ***Syzygium wilsonii*** | **Myrtaceae** | **Evergreen** | **Tree** | **Rainforest** | **Tropical** | **HIE** |
| ***Xylosma congesta*** | **Salicaceae** | **Evergreen** | **Tree** | **Forest margin** | **Subtropical** | **HIE** |
| *Acacia brachybotrya* | Fabaceae | Evergreen | Shrub | Mallee woodland | Arid | MQ |
| ***Alyogyne huegelii*** | **Malvaceae** | **Evergreen** | **Shrub** | **Mallee woodland** | **Arid** | **MQ** |
| ***Angophora costata*** | **Myrtaceae** | **Evergreen** | **Tree** | **Coastal woodland** | **Subtropical** | **MQ** |
| ***Betula utilis*** | **Betulaceae** | **Evergreen** | **Tree** | **Subalpine forest** | **Temperate** | **MQ** |
| ***Elaeocarpus reticulatus*** | **Elaeocarpaceae** | **Evergreen** | **Tree** | **Eucalypt forest** | **Subtropical** | **MQ** |
| *Eremophila maculata* | Scrophulariaceae | Evergreen | Shrub | Open woodland, scrubland | Arid | MQ |
| ***Erythrina vespertilio*** | **Fabaceae** | **Deciduous** | **Tree** | **Open woodland** | **Tropical** | **MQ** |
| ***Geijera parviflora*** | **Rutaceae** | **Evergreen** | **Shrub** | **Dry sclerophyll forest** | **Arid** | **MQ** |
| *Hydrangea macrophylla* | Hydrangeaceae | Deciduous | Shrub | Wet forest | Temperate | MQ |
| ***Lagerstroemia indica*** | **Lythraceae** | **Deciduous** | **Tree** | **Forest margin** | **Subtropical** | **MQ** |
| *Liquidambar styraciflua* | Altingiaceae | Deciduous | Tree | Deciduous forest | Temperate | MQ |
| *Melastoma affine* | Melastomataceae | Evergreen | Tree | Wet sclerophyll forest | Tropical | MQ |
| ***Melia azedarach*** | **Meliaceae** | **Evergreen** | **Tree** | **Rainforest** | **Subtropical** | **MQ** |
| ***Notelaea microcarpa*** | **Oleaceae** | **Evergreen** | **Tree** | **Dry sclerophyll woodland** | **Temperate** | **MQ** |
| ***Photinia* × *robusta*** | **Rosaceae** | **Evergreen** | **Shrub** | **N/A (hybrid)** | **Temperate** | **MQ** |
| ***Pistacia chinensis*** | **Anacardiaceae** | **Deciduous** | **Tree** | **Montane forest** | **Subtropical** | **MQ** |
| ***Platanus* × *acerifolia*** | **Platanaceae** | **Deciduous** | **Tree** | **N/A (hybrid)** | **Temperate** | **MQ** |
| ***Prunus cerasifera*** | **Rosaceae** | **Deciduous** | **Tree** | **Open forest** | **Temperate** | **MQ** |
| ***Pyrus calleryana*** | **Rosaceae** | **Deciduous** | **Tree** | **Open forest** | **Temperate** | **MQ** |
| ***Quercus palustris*** | **Fagaceae** | **Deciduous** | **Tree** | **Deciduous forest** | **Temperate** | **MQ** |
| ***Quercus robur*** | **Fagaceae** | **Deciduous** | **Tree** | **Deciduous forest** | **Temperate** | **MQ** |
| ***Rhaphiolepis umbellata*** | **Rosaceae** | **Deciduous** | **Shrub** | **Coastal forest** | **Subtropical** | **MQ** |
| *Toona ciliata* | Meliaceae | Deciduous | Tree | Rainforest | Subtropical | MQ |
| ***Viburnum tinus*** | **Adoxaceae** | **Evergreen** | **Shrub** | **Mediterranean woodland** | **Subtropical** | **MQ** |

Table S2. Mean midday values of photosynthetically active radiation (PAR), relative humidity, and air temperature at the two glasshouse locations: Hawkesbury Institute for the Environment (HIE) and Macquarie University (MQ). Reported values are the mean minimum, mean and mean maximum daily values at midday (12:00-14:00) averaged for baseline and +7 °C heatwave conditions throughout the course of the study. Values shown are aggregated across all plant sampling batches at each glasshouse location.

| Location/ Treatment | PAR [μmol m^-2^ s^-1^] | | | Relative Humidity [%] | | | Temperature [°C] | | |
| --- | --- | --- | --- | --- | --- | --- | --- | --- | --- |
|  | Min | Mean | Max | Min | Mean | Max | Min | Mean | Max |
| HIE Baseline | 263 | 1007 | 2197 | 56 | 63 | 70 | 33.2 | 35.0 | 36.2 |
| HIE Heatwave | 220 | 844 | 1864 | 51 | 60 | 69 | 38.1 | 40.6 | 42.0 |
| MQ Baseline | 386 | 840 | 1228 | 60 | 63 | 68 | 31.6 | 33.5 | 35.1 |
| MQ Heatwave | 714 | 999 | 1221 | 53 | 56 | 61 | 37.7 | 40.8 | 43.1 |

Table S3. Diurnal air temperature profile for the baseline and +7 °C heatwave treatment applied at two glasshouse locations in this study.

| **Time** | **Baseline temperature [°C]** | **Heatwave temperature [°C]** |
| --- | --- | --- |
| 00:00-06:00 | 21 | 30 |
| 06:00-08:00 | 27 | 34 |
| 08:00-10:00 | 30 | 36 |
| 10:00-12:00 | 32 | 38 |
| 12:00-14:00 | 34 | 41 |
| 14:00-18:00 | 34 | 40 |
| 18:00-20:00 | 29 | 36 |
| 20:00-00:00 | 21 | 30 |
| Minimum | 21 | 30 |
| Maximum | 34 | 41 |
|  |  |  |

Table S4. Species mean (±SE) critical temperature (*T_crit_*), maximum leaf temperature (*T_leaf_max_*) and the thermal safety margin (*TSM*) of 50 tree or shrub species under baseline and +7 °C heatwave conditions. Asterisks denote significant changes in heatwave *T_crit_* and *TSM*, according to Student’s *t*-tests: *** *p*<0.001, ** *p*<0.01, * *p*<0.05, and • *p*<0.1. The *TSM* are shown for a subset of 34 tree and shrub species and were dependent upon measurement of *T_leaf_max_* under both baseline and heatwave conditions (*n*=5 plants per species); species with *TSM* values <7 °C are shown in bold.

| **Species** | ***T_crit_*** | | ***T_leaf_max_*** | | ***TSM*** | |
| --- | --- | --- | --- | --- | --- | --- |
|  | **Baseline** | **Heatwave** | **Baseline** | **Heatwave** | **Baseline** | **Heatwave** |
| *Acacia brachybotrya* | 47.2 ± 0.3 | 46.2 ± 0.5 | NA | NA | NA | NA |
| *Acronychia oblongifolia* | 50.0 ± 0.1 | 51.2 ± 1.1 | NA | NA | NA | NA |
| *Agonis flexuosa* | 47.2 ± 0.2 | 51.3 ± 0.5** | NA | NA | NA | NA |
| *Alnus jorullensis* | 47.0 ± 0.9 | 48.6 ± 0.4 | 36.1 ± 1.0 | 47.7 ± 1.2 | 10.6 ± 1.1 | 0.9 ± 1.3** |
| *Alphitonia excelsa* | 49.7 ± 0.6 | 51.1 ± 0.3 | NA | NA | NA | NA |
| *Alyogyne huegelii* | 48.1 ± 0.5 | 48.3 ± 0.2 | 31.8 ± 0.3 | 36.2 ± 0.2 | 16.5 ± 0.5 | 12.2 ± 0.4*** |
| *Angophora costata* | 47.0 ± 0.4 | 49.7 ± 0.5** | 32.4 ± 1.2 | 39.7 ± 0.8 | 15.4 ± 0.7 | 9.9 ± 0.8** |
| ***Atractocarpus benthamianus*** | **50.3 ± 0.6** | **47.5 ± 0.5*** | **45.2 ± 0.9** | **52.9 ± 1.0** | **5.1 ± 0.6** | **-5.4 ± 0.5**** |
| ***Atractocarpus fitzalanii*** | **50.5 ± 0.6** | **52.9 ± 0.7*** | **45.3 ± 1.1** | **51.1 ± 1.5** | **5.2 ± 0.6** | **1.9 ± 0.7** |
| *Banksia robur* | 48.7 ± 0.3 | 48.7 ± 0.7 | 40.8 ± 0.6 | 46.8 ± 0.3 | 7.9 ± 0.8 | 1.9 ± 0.9** |
| *Banksia serrata* | 47.3 ± 0.3 | 49.3 ± 0.7• | 33.5 ± 0.3 | 46.1 ± 0.9 | 13.8 ± 0.5 | 2.9 ± 1.4** |
| *Betula utilis* | 47.3 ± 0.2 | 47.8 ± 0.3 | 30.9 ± 0.2 | 35.3 ± 0.3 | 16.4 ± 0.2 | 12.5 ± 0.3*** |
| *Brachychiton populneus* | 49.5 ± 0.3 | 52.0 ± 0.5** | NA | NA | NA | NA |
| *Brachychiton rupestris* | 49.3 ± 0.7 | 51.0 ± 1.0 | NA | NA | NA | NA |
| *Callistemon citrinus* | 48.1 ± 0.4 | 48.7 ± 0.5 | 34.8 ± 0.9 | 42.2 ± 0.9 | 13.5 ± 1.3 | 7.7 ± 0.6* |
| *Ceratopetalum apetalum* | 49.8 ± 0.2 | 48.9 ± 0.9 | NA | NA | NA | NA |
| *Elaeocarpus reticulatus* | 48.5 ± 0.2 | 50.3 ± 0.4** | 32.4 ± 0.4 | 36.1 ± 0.3 | 16.1 ± 0.6 | 14.3 ± 0.5* |
| *Eremophila bignoniiflora* | 48.5 ± 0.3 | 48.8 ± 0.6 | 34.9 ± 0.4 | 40.1 ± 0.9 | 13.4 ± 0.7 | 8.7 ± 1.7• |
| *Eremophila maculata* | 46.7 ± 0.2 | 48.7 ± 0.3*** | NA | NA | NA | NA |
| *Erythrina vespertilio* | 45.7 ± 0.4 | 47.1 ± 0.4* | 31.8 ± 0.8 | 37.5 ± 0.5 | 13.9 ± 1.3 | 9.7 ± 0.8* |
| *Eucalyptus populnea* | 47.6 ± 0.4 | 51.6 ± 0.4*** | 38.7 ± 1.1 | 45.7 ± 1.0 | 9.5 ± 0.9 | 5.5 ± 1.3* |
| *Eucryphia lucida* | 47.3 ± 0.4 | NA | NA | NA | NA | NA |
| ***Flindersia brayleyana*** | **49.7 ± 0.8** | **50.2 ± 0.6** | **45.2 ± 0.9** | **49.5 ± 0.5** | **3.7 ± 0.8** | **0.7 ± 0.6*** |
| ***Flindersia xanthoxyla*** | **49.9 ± 0.2** | **52.7 ± 0.4**** | **46.2 ± 0.6** | **50.7 ± 1.2** | **3.7 ± 0.2** | **2 ± 0.4** |
| *Geijera parviflora* | 47.4 ± 0.4 | 48.8 ± 0.2* | 31.3 ± 0.2 | 36.1 ± 0.6 | 16.1 ± 0.4 | 12.6 ± 0.7** |
| *Hydrangea macrophylla* | 46.8 ± 0.3 | 49.2 ± 0.3** | NA | NA | NA | NA |
| *Lagerstroemia indica* | 47.2 ± 0.2 | 50.1 ± 0.5** | 28.9 ± 0.4 | 37.0 ± 0.2 | 18.3 ± 0.3 | 12.9 ± 0.7** |
| *Liquidambar styraciflua* | 48.3 ± 0.4 | 47.6 ± 0.6 | NA | NA | NA | NA |
| *Melastoma affine* | 47.4 ± 0.4 | 47.1 ± 0.6 | NA | NA | NA | NA |
| *Melia azedarach* | 49.0 ± 0.5 | 49.3 ± 0.4 | 35.4 ± 0.7 | 43.0 ± 0.6 | 13.6 ± 1.0 | 6.1 ± 0.6*** |
| *Notelaea microcarpa* | 47.8 ± 0.4 | 48.3 ± 0.2 | 32.1 ± 0.4 | 35.9 ± 0.4 | 15.7 ± 0.5 | 12.4 ± 0.3** |
| *Photinia robusta* | 49.1 ± 0.4 | 49.6 ± 0.3 | 34.3 ± 0.8 | 40.56 ± 0.8 | 14.8 ± 0.9 | 9.1 ± 0.8** |
| *Pistacia chinensis* | 48.2 ± 0.3 | 49.5 ± 0.4* | 36.0 ± 0.2 | 40.0 ± 0.6 | 12.6 ± 0.2 | 9.5 ± 0.8* |
| *Pittosporum angustifolium* | 48.0 ± 0.7 | 49.6 ± 0.9 | NA | NA | NA | NA |
| *Pittosporum bicolor* | 45.7 ± 0.8 | NA | NA | NA | NA | NA |
| *Platanus* x *acerifolia* | 49.3 ± 0.4 | 50.1 ± 0.4 | 29.6 ± 0.3 | 39.0 ± 0.2 | 19.7 ± 0.4 | 11.1 ± 0.5*** |
| *Podocarpus elatus* | 48.7 ± 0.7 | 50.7 ± 0.6• | NA | NA | NA | NA |
| *Pomaderris apetala* | 47.8 ± 0.8 | 51.0 ± 0.8* | 39.9 ± 0.5 | 49.3 ± 0.6 | 8.2 ± 0.9 | 1.6 ± 1.5* |
| *Prunus cerasifera* | 47.7 ± 0.4 | 49.5 ± 0.6• | 33.3 ± 0.5 | 39.4 ± 1.4 | 14.4 ± 0.5 | 11.2 ± 0.9* |
| *Pyrus calleryana* | 47.2 ± 0.4 | 49.4± 0.5* | 31.6 ± 0.8 | 40.2 ± 1.1 | 16.0 ± 0.7 | 9.2 ± 1.3** |
| *Quercus palustris* | 46.9 ± 0.6 | 49.0 ± 0.5* | 31.7 ± 0.5 | 38.7 ± 0.9 | 15.2 ± 0.6 | 10.3 ± 0.54*** |
| *Quercus robur* | 48.4 ± 0.5 | 48.0 ± 0.2 | 32.7 ± 1.1 | 33.8 ± 1.5 | 15.7 ± 0.8 | 14.2 ± 1.33 |
| *Rhaphiolepis umbellata* | 47.3 ± 0.2 | 49.1 ± 0.3** | 32.5 ± 0.5 | 41.1 ± 0.4 | 14.8 ± 0.5 | 8.0 ± 0.23*** |
| ***Stenocarpus sinuatus*** | **48.3 ± 0.4** | **51.4 ± 0.3**** | **45.9 ± 1.2** | **52.4 ± 0.4** | **3.1 ± 1.6** | **-0.8 ± 0.48**• |
| ***Syzygium anisatum*** | **49.2 ± 0.4** | **47.4 ± 1.9** | **44.2 ± 1.0** | **50.1 ± 1.2** | **5.0 ± 0.4** | **-3.9 ± 1.93**• |
| *Syzygium smithii* | 46.9 ± 0.4 | 49.4 ± 0.3** | 38.8 ± 0.3 | 49.4 ± 1.1 | 8.0 ± 0.2 | 0.03 ± 1.11** |
| ***Syzygium wilsonii*** | **48.8 ± 0.1** | **47.3 ± 0.6•** | **42.4 ± 0.6** | **48.6 ± 1.1** | **6.4 ± 0.1** | **-1.3 ± 0.59**** |
| *Toona ciliata* | 47.1 ± 0.4 | 47.3 ± 0.5 | NA | NA | NA | NA |
| *Viburnum tinus* | 47.3 ± 0.3 | 48.8 ± 0.4* | 32.5 ± 0.5 | 38.0 ± 0.3 | 14.8 ± 0.4 | 10.8 ± 0.63** |
| *Xylosma congesta* | 49.5 ± 0.7 | 51.1 ± 0.4• | 39.5 ± 1.3 | 46.9 ± 0.5 | 10.7 ± 2.1 | 3.8 ± 0.35• |

Figure S1. Relationships between heat tolerance (*T_crit_*) and (A) maximum leaf temperature (*T_leaf_max_*) in the HIE: *T_crit_*= 42.08 + 0.16 * *T_leaf_max_* (*r^2^*=0.29, *p*=0.001, *n*=30) and MQ glasshouses: *T_crit_*= 41.28 + 0.20 * *T_leaf_max_* (*r^2^*=0.45, *p*<0.001, *n*=34), and (B) the difference between *T_leaf_max_* and air temperature (*T_diff_*) of 34 tree or shrub species: *T_crit_* = 48.15 + 0.17 * *T_diff_* (*r^2^*=0.18, *p*=0.014, *n*=30), as shown in Figure 2 but here shown for two different glasshouse locations (HIE and MQ). ANCOVA showed no difference in *T_crit_* (*p*=0.25) but significant differences in *T_leaf_max_* (*Χ^2^*=14.06, *p*<0.001) and *T_diff_* (*Χ^2^*=16.67, *p*<0.001) between the two locations.

Figure S2. Change in heat tolerance (Δ*T_crit_*) for 48 tree or shrub species in response to a six-day +7 °C heatwave, compared to baseline growing conditions. The species are ordered according to their mean Δ*T_crit_* value (*n*= 3-5 plants per species); error bars represent SE. Positive values indicate an increase in heat tolerance after exposure to 41 °C heatwave. Two species (*Eucryphia lucida*, *Pittosporum bicolor*) are missing due to leaf loss during the heatwave, precluding calculation of Δ*T_crit_*.

Figure S3. (A) Maximum quantum yield of PS II (*F_v_/F_m_*) and (B) leaf visible injury were nonlinearly related to maximum leaf temperature (*T_leaf_max_*) under +7 °C heatwave conditions in 34 tree or shrub species. In (A), a generalized additive model of heatwave *F_v_/F_m_* and heatwave *T_leaf_max_* was used (*F*_3,3.7_=6.49, *r^2^*=0.41, *p*=0.001). In (B), a generalized additive model of leaf visible injury and *T_leaf_max_* was used (*F*_1,1_=9.18, *r^2^*=0.20, *p*=0.005). Values are species means (*n*=3-5 plants per species); error bars represent SE.
